# Putative Anticancer Compounds from Plant-Derived Endophytic Fungi: A Review

**DOI:** 10.3390/molecules27010296

**Published:** 2022-01-04

**Authors:** Md. Hridoy, Md. Zobayer Hossain Gorapi, Sadia Noor, Nargis Sultana Chowdhury, Md. Mustafizur Rahman, Isabella Muscari, Francesco Masia, Sabrina Adorisio, Domenico V. Delfino, Md. Abdul Mazid

**Affiliations:** 1 Department of Pharmacy, University of Dhaka, Dhaka 1000, Bangladesh; md.hridoy@temple.edu; 2 Department of Pharmaceutical Sciences, School of Pharmacy, Temple University, Philadelphia, PA 19140, USA; 3 Department of Pharmacy, University of Asia Pacific, Dhaka 1215, Bangladesh; mdzobayerhossain14@gmail.com (M.Z.H.G.); noorsadia.du@uap-bd.edu (S.N.); 4 Department of Pharmaceutical Chemistry, Faculty of Pharmacy, University of Dhaka, Dhaka 1000, Bangladesh; 5 Department of Pharmacy, Manarat International University, Dhaka 1343, Bangladesh; nargis@manarat.ac.bd; 6 Pharmacy Discipline, Khulna University, Khulna 9208, Bangladesh; mmrahman0103@pharm.ku.ac.bd; 7 Department of Medicine and Surgery, University of Perugia, 06132 Perugia, Italy; isa.muscari2@gmail.com (I.M.); francesco.masia@unipg.it (F.M.); 8 Department of Medicine and Surgery, Foligno Nursing School and Section of Pharmacology, University of Perugia, Piazzale Severi, S. Andrea delle Fratte, 06129 Perugia, Italy; adorisiosabrina@libero.it

**Keywords:** endophytic fungi, anticancer compounds, living plants

## Abstract

Endophytic fungi are microorganisms that exist almost ubiquitously inside the various tissues of living plants where they act as an important reservoir of diverse bioactive compounds. Recently, endophytic fungi have drawn tremendous attention from researchers; their isolation, culture, purification, and characterization have revealed the presence of around 200 important and diverse compounds including anticancer agents, antibiotics, antifungals, antivirals, immunosuppressants, and antimycotics. Many of these anticancer compounds, such as paclitaxel, camptothecin, vinblastine, vincristine, podophyllotoxin, and their derivatives, are currently being used clinically for the treatment of various cancers (e.g., ovarian, breast, prostate, lung cancers, and leukemias). By increasing the yield of specific compounds with genetic engineering and other biotechnologies, endophytic fungi could be a promising, prolific source of anticancer drugs. In the future, compounds derived from endophytic fungi could increase treatment availability and cost effectiveness. This comprehensive review includes the putative anticancer compounds from plant-derived endophytic fungi discovered from 1990 to 2020 with their source endophytic fungi and host plants as well as their antitumor activity against various cell lines.

## 1. Introduction

In 1866, de Bary introduced the term “endophyte” [1]. An endophyte may be a fungal or bacterial microorganism that colonizes various interior parts of plants causing no apparent pathogenic effects on its host plants. The endophytes, most commonly endophytic fungi, are believed to help plants adapt to abiotic factors (high temperature and salinity, drought, metal toxicity, and harmful effects of light) as well as biotic factors (herbivores, insects, nematodes, and pathogens). This is mainly achieved by the secondary bioactive metabolites produced by the endophytic fungi. In their symbiotic relation, the endophytes are fed and protected by the host plant, and in return, these microorganisms produce bioactive secondary metabolites, enhancing the growth of the host plant and protecting the plant from pathogens and herbivores [2]. Therefore, endophytic fungal metabolites can also be exploited as drugs for the treatment of various types of human diseases, including cancer [3].

This group of microorganisms has drawn tremendous attention from researchers since the isolation, culture, purification, and characterization of this fascinating group of microorganisms revealed the presence of hundreds of important and diverse chemical classes of compounds. The interest of scientists in endophytes is also growing as they are a good reservoir of bioactive metabolites [4,5]. Until now, many cytotoxic agents including paclitaxel (also known as Taxol) [6] have been isolated from endophytes. Secondary metabolites with cytotoxic properties have the potential to be explored as anticancer drugs.

Recent studies revealed that naphthoquinone derivatives fusarubins including anhydrofusarubin and fusarubin (FUS) produced by endophytic fungi *Cladosporium* species [7] and *Fusarium* species [8] showed promising cytotoxicity against cancer cells. Although FUS was reported earlier to have antibacterial activity, its cytotoxic activity was reported recently. Very recently, for the first time, we have revealed the molecular mechanism of cytotoxic action of fusarubin isolated from a *Cladosporium* species inhabiting the leaves of *Rauwolfia serpentina*. We have reported that fusarubin and anhydrofusarubin inhibit proliferation and increase apoptosis in leukemia and other hematological tumor cells lines in different manners through the p21/p53-mediated pathway [9]. Our findings urge us to write this review on endophytic fungal metabolites as a fascinating group of bioactives or putative anticancer compounds. Many of these putative anticancer compounds have very promising cytotoxicity against a broad spectrum of cancer cell lines; some compounds are already used as treatments for different cancer types such as breast, bladder, colorectal, esophageal, lung, ovarian, prostate, melanoma, testicular, leukemia, and lymphoma.

## 2. Anticancer Activity of Endophytic Fungi

**Figure 1:**
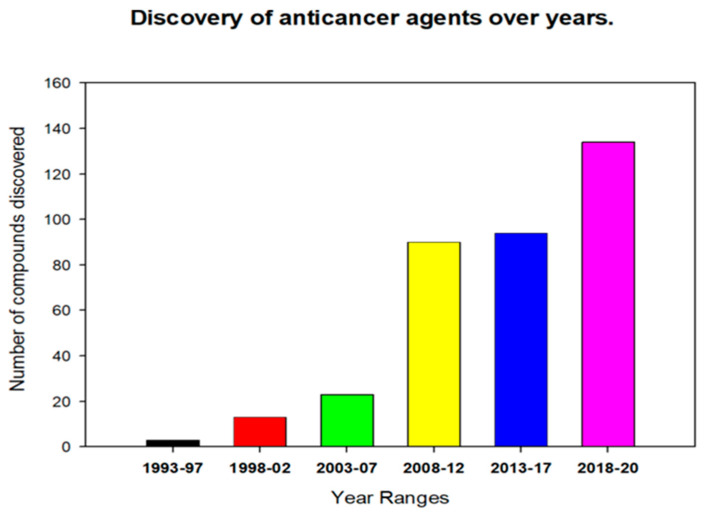
Discovery of anticancer agents from endophytic fungi over time.

**Figure 2:**
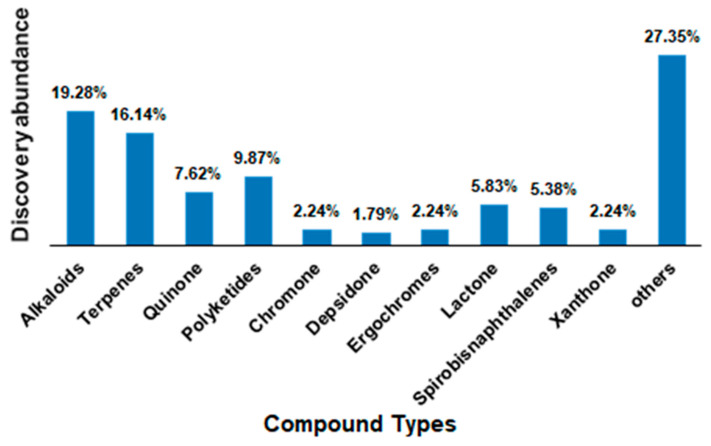
Relative abundance of anticancer agents from endophytic fungi.

Endophytic fungi have been a known source of anticancer agents since the discovery of the valuable drug Taxol (also known as paclitaxel, a diterpenoid) isolated for the first time from an endophytic fungus *Taxomyces andreanae* obtained from the Pacific Yew bark (*Taxus brevifolia*) [6]. Since then, other anticancer drugs have been isolated from endophytic fungi, and among these 9-methoxycamptothecin and 10-hydroxycamptothecin from *Fusarium solani* [10], camptothecin from *Entrophospora infrequens* [11]; the anticancer lead compounds podophyllotoxin from *Phialocephala fortinii* [12] and deoxypodophyllotoxin from *Aspergillus fumigatus* [13] fueled further research on endophytic fungi to discover many other important known and novel anticancer compounds. According to this review, until now, more than 100 different fungal species have been identified to produce more than two hundred putative anticancer compounds (Figure 1 and Figure 2) reported to possess antiproliferative and/or cytotoxic properties against more than 60 different cell lines (Table 1, Table 2 and Table 3). Figure 1 indicates that endophytic fungal-derived anticancer agents gained attention from scientists over the past three decades. Meanwhile, Figure 2 represents the abundance of different chemical classes and diversity of fungal metabolites. The anticancer compounds isolated from endophytic fungi are effective against diverse cell lines that could be helpful in combating any particular type of cancer (Table 1).

The genera of endophytic fungi containing two or more putative anticancer-agent-producing species are *Acremonium*, *Alternaria*, *Aspergillus*, *Ceriporia*, *Chaetomium*, *Colletotrichum*, *Cytospora*, *Emericella*, *Eurotium*, *Eutypella*, *Fusarium*, *Guignardia*, *Hypocrea*, *Penicillium*, *Pestalotiopsis*, *Phomposis*, *Periconia*, *Stemphylium*, *Talaromyces*, *Thielavia*, and *Xylaria* [4,221]. These endophytic fungi offer an alternative source of bioactive compounds. We may be able to increase their yield of specific anticancer compounds by employing biotechnology and genetic engineering [221].

### 2.1. Anti-Cancer Agents in Clinical Use Shared by Plants and Endophytic Fungi

**Figure 3:**
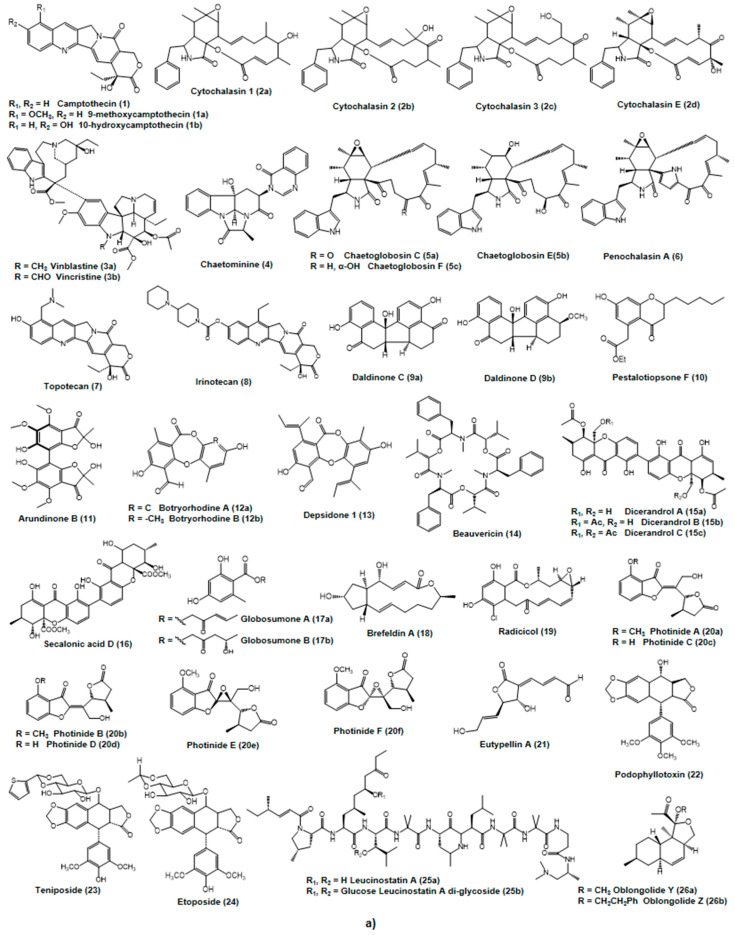

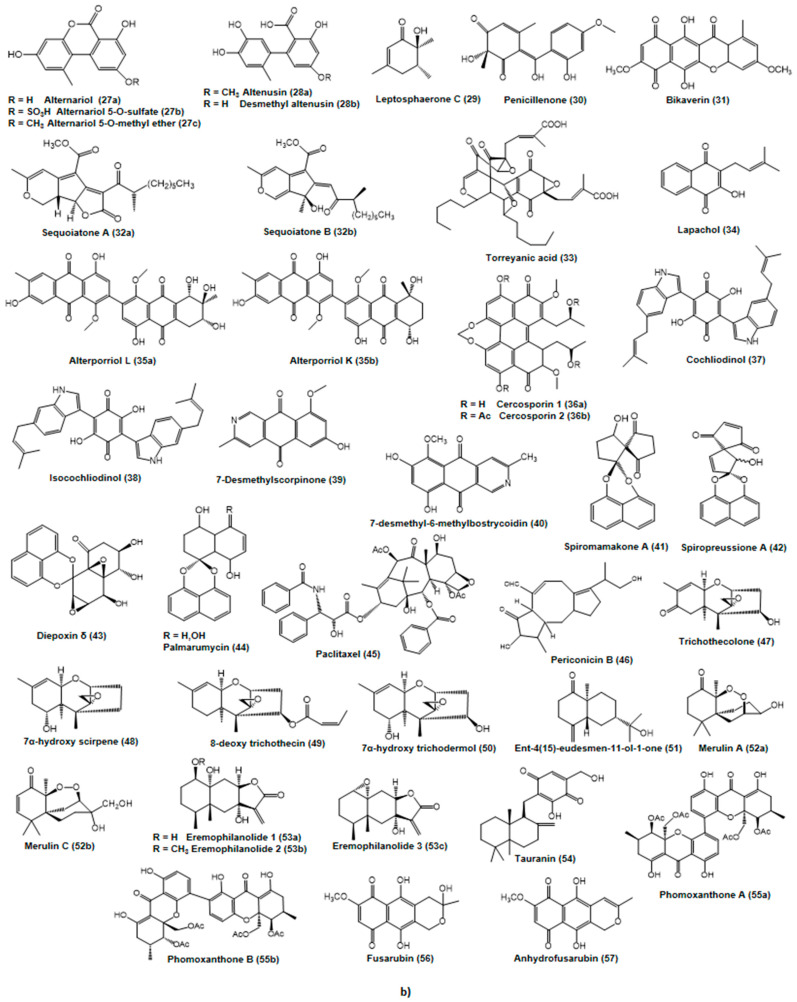
Anticancer compounds of different chemical classes from endophytic fungi-alkaloidal compounds and their derivatives: (**a**) (1–8), benzo[j]fluoranthene (9), Chromone (10), coumarin (11), depsidones (12, 13), depsideptide (14), ergochromes (15, 16), ester (17), lactones (18–22), lignans (23–24), peptide (25), polykedites (26); (**b**) polyketides (27–32), quinones (33–39), spirobisnaphthalenes (40–42), terpenes (43–54), xanthones (55), naphthoquinones (56, 57).

Plants are prolific sources of anticancer agents. In the area of cancer, of the 175 approved small molecules over the years from the 1940s to 2014, 75% (131) are other than synthetic and 49% (85) are either natural products or their derivatives [222]. Very recently, it was reported that among the approved 321 anticancer molecules from all sources during the period of 1946 to 2019, 35 (10.9%) were unaltered natural products and 65 (20.2%) were natural product derivatives compared to 53 (16.5%) completely synthetic drug molecules. Some of these agents obtained from plants are also found in their corresponding endophytic fungi. The following are some examples of plant/endophytic fungi-derived cancer effective agents [1,6] (Figure 3a,b).

Paclitaxel (Taxol^®^) is used in combination with other anti-cancer drugs in ovarian, breast, non-small cell lung cancer (NSCLC), and Kaposi sarcoma. An active paclitaxel analogue, docetaxel is used in breast and non-small cell lung cancer (NSCLC) treatment [223]. Even though camptothecin exerted severe bladder toxicity in its clinical trial in the 1970s and therefore, was dropped, its two water-soluble derivatives, topotecan and irinotecan, have been shown to be more effective anti-cancer agents and are being utilized for these purposes [223]. Topotecan (Hycamtin^®^) was the first CPT derivative that was orally available and has been approved for cervical (when used in combination with cisplatin), ovarian, and non-small cell lung cancer treatment. Irinotecan (Camptosar^®^) has been approved for colorectal cancer treatment. These agents show cytotoxicity on account of their ability to inhibit a fundamental enzyme, topoisomerase-I, involved in the winding and unwinding process of DNA during replication or protein synthesis [1,223]. The vinca alkaloids, vinblastine and vincristine, and their semi-synthetic analogs, vinorelbine and vindesine, are primarily used in combination with other chemotherapeutic drugs in the treatment of advanced testicular cancer, breast cancer, Kaposi’s sarcoma, lung cancer, leukemias, and lymphomas [223]. Etoposide and teniposide are clinically effective semi-synthetic derivatives of a podophyllotoxin isomer, epipodophyllotoxin, which are used in bronchial cancers, lymphomas, and testicular cancer treatments [223].

### 2.2. Putative Anticancer Compounds from Endophytic Fungi

#### 2.2.1. Alkaloids and Nitrogen-Containing Heterocycles

Camptothecin (CPT) (1), a pentacyclic quinoline alkaloid, was, at first, isolated from the *Camptotheca *acuminata** (happy tree) woods showing antileukemic and anti-cancer effects in animals [1]. It exerts its cytotoxicity by inhibition and dissociation of the DNA-topoisomerase-I complex during DNA replication [224,225]. However, recently, CPT has been isolated from some endophytic fungi, *Entrophospora i.*, residing in these plants. Since *Entrophospora i.* also lives inside the inner bark of *Nothapodytes foetida* [11], in 2008, CPT was isolated from a *Nothapodytes foetida* seed endophyte, *Neurospora c.*, and both authentic and fungal CPT exhibited comparable cytotoxic effects in human cancer cell lines HEP-2 (liver cancer), A549 (lung cancer), and OVCAR-5 (ovarian cancer) [96]. In 2009, CPT along with its two derivatives, 9-methoxycamptothecin and 10-hydroxycamptothecin, were isolated from a *Camptotheca acuminata* inner bark endophyte, *Fusarium s.* (Figure 3a). These derivatives are more water soluble and more potent inhibitors of the topoisomerase-I enzyme [80] (Table 2).

Cytochalasins (2a–2d) are fungal metabolites that inhibit cell division by means of inhibiting actin filament polymerization [226]. Four cytochalasins (cytochalasin 1, 2, 3, and E) have been isolated from an endophytic fungus, *Rhinocladiella* spp. from the *Tripterygium wilfordii* dead tree limbs and were tested against HCT-116 (colon tumor cell line), A2780S (ovarian tumor cell line), and SW-620 (colon tumor cell line) showing cytotoxic activities [136].

The vinca alkaloid (3a, 3b), vincristine (leurocristine), was isolated from *Catharanthus roseus* [227]. This alkaloid has also been isolated from some fungal endophytes of *Catharanthus roseus* such as *Fusarium o.* (inner bark), *Mycelia s.* 97CY(3) (Leaves), and *Talaromyces r.* CrP20 (Leaves) [74,75,89]. Vincristine irreversibly binds to the spindle proteins and microtubules during the S-phase of cell cycle hampering mitotic spindle formation and therefore arresting tumor cell division in the metaphase [1].

Chaetominine (4) was isolated from an endophyte, *Chaetomium* sp. IFB-E015 from the healthy leaves of *Adenophora axilliflora*, and it was cytotoxic against K562 (human leukemia cells) and SW1116 (human colon cancer cells) [54].

Cytochalasan-based alkaloids (5a–5c, 6), namely chaetoglobosin C, E, F, U, and penochalasin A (6), were obtained from the endophyte *Chaetomium g.* IFB-E019 residing inside the *Imperata cylindrica* healthy stem. Chaetoglobosin U was cytotoxically active against the KB cell line (human nasopharyngeal epidermoid tumor) with an IC50 value of 16.0 µM, whereas chaetoglobosin C (IC50 34.0 µM), E (IC50 40.0 µM), F (IC50 52.0 µM), and penochalasin A (IC50 48.0 µM) were moderately active against the KB cell line [57]. Endophytic fungus *Chaetomium g.* L18 from the plant *Curcuma wenyujin* produces chaetoglobosin X that exerted cytotoxic activity against H22 (hepatic cancer cells in mice) and MFC (gastric cancer cells in mice) cell lines [56] (Table 2).

#### 2.2.2. Benzo[j]fluoranthenes

Daldinone C (9a) and D (9b) were discovered from an *Artemisia Artemisia annua* endophyte, *Hypoxylon t.* IFB-18, where both agents exerted strong cytotoxic action against the human colorectal cancer SW1116 cell line at IC50 values of 49.5 and 41.0 μM, respectively [85] (Table 2, Figure 3a).

#### 2.2.3. Chromones

A novel chromone, Pestalotiopsone F (10), was isolated from an endophytic fungus *Pestalotiopsis* spp. associated with a mangrove plant *Rhizophora mucronata*. Pestalotiopsone F showed moderate cytotoxicity to L5178Y (murine cancer cell line) at an EC50 value of 8.93 μg/mL [110]. Pestaloficiol I, J, K, and L are new isoprenylated chromone derivatives discovered from a *Camellia sinensis* endophyte, Pestalotiopsis f., that displayed cytotoxicity against HeLa (Cervical cancer) and MCF-7 (Breast cancer) cell lines [115] (Table 2).

#### 2.2.4. Coumarins

Arundinone B (11) was isolated from an endophyte *Microsphaeropsis a*. associated with *Ulmus macrocarpa*. The compound showed cytotoxicity to T24 (Bladder carcinoma) and A549 (Lung carcinoma epithelial) cell lines [92] (Table 2).

#### 2.2.5. Depsidones

Botryorhodines A (12a) and B (12b), two depsidones, were isolated from the endophytic fungus *Botryosphaeria r.* associated with *Bidens pilosa*. These compounds exhibited weak antitumor activity against the HeLa cell line at a concentration of 96.97 and 36.41 μM, respectivel [48]. Depsidone 1 was discovered from a fungus of the Pleosporales order (BCC 8616) isolated from an unidentified plant leaf of the Hala-Bala forest origin. Depsidone 1 displayed weak cytotoxicity to KB and BC cell lines with IC50 values 6.5 and 4.1μg/mL, respectively [43] (Table 2).

#### 2.2.6. Depsipeptides

Beauvericin (14), a depsipeptide, was isolated from two fungi, *Fusarium o*. EPH2RAA and *Fusarium o*., associated with the plants *Cylindropuntia echinocarpus* and *Ephedra fasciculate*, respectively. Beauvericin displayed cytotoxicity to NCI-H460 (human non-small cell lung cancer), MIA Pa Ca-2 (human pancreatic carcinoma), MCF-7 (human breast cancer), and SF-268 (human CNS cancer) cell lines with IC50 values of 1.41, 1.66, 1.81, and 2.29 μM, respectively, showing selective cytotoxicity toward MIA PaCa-2 and NCI-H460 (Table 2). Beauvericin also inhibited the metastasis of MDA-MB-231 (Breast cancer) and PC-3M (metastatic prostate cancer) cells at concentrations ranging between 3.0–4.0 and 2.0–2.5 µM, respectively [77]. According to other studies, beauvericin displayed cytotoxicity against A549 (Lung carcinoma epithelial), PC-3 (Prostate cancer), and PANC-1 (human pancreatic carcinoma) cell lines with IC50 values of 10.4 ± 1.6, 49.5 ± 3.8, and 47.2 ± 2.9 μM, respectively [71]. Additionally, in 2006, Ivanova et al. demonstrated the cytotoxicity of beauvericin against Hep-G2 (hepatocellular carcinoma) and MRC-5 (fibroblast-like fetal lung cell line) cells as well [76].

#### 2.2.7. Ergochromes

*Phomopsis l.*, an endophytic fungus of *Dicerandra frutescens*, produced three compounds dicerandrols A, B, and C (15a–15c), structurally related to the ergochromes and secalonic acids as they also have the same tricyclic C15 system with a similar arrangement of substituents. These compounds displayed modest antitumor activities toward A549 (lung adenocarcinoma epithelial cell line) and HCT-116 (colon tumor cell line) cell lines [132] (Table 2).

Secalonic acid D (16), isolated from mangrove plant endophytic fungus no. ZSU44, displayed potent cytotoxicity against HL60 (the human promyelocytic leukemia cell line) and K562 (human leukemia cells) cells with IC50 values of 0.38 and 0.43 μM, respectively. It caused apoptosis in those cell lines and cell cycle arrest in the G(1) phase as well [158].

#### 2.2.8. Esters

Globosumones A (17a) and B (17b), isolated from the endophyte *Chaetomium g*. associated with *Ephedra fasciculate*, were shown to have cytotoxicity to MCF-7 (breast cancer), MIA PaCa-2 (pancreatic carcinoma), NCI-H460 (non-small cell lung cancer), SF-268 (CNS glioma), and WI-38 (normal human fibroblast cells) cell lines [58].

#### 2.2.9. Lactones

The lactone compound Brefeldin A (18) was obtained from two endophytic fungi, *Aspergillus c*. and *Paecilomyces* spp., isolated from the plants *Taxus mairei* and *Torreya grandis*. Brefeldin A exhibited antitumor activities to Hela, HL-60, KB, MCF-7, and Spc-A-1 with IC50 values of 1.8, 10.0, 9.0, 2.0, and 1.0 ng/mL [31]. Brefeldin A was also obtained from the endophyte *Acremonium* spp. isolated from the healthy *Knema laurina* twig. It showed cytotoxicity to BC-1 (breast cancer), KB (epidermoid cancer of the mouth), and NCIH187 (human small-cell lung cancer), with IC50 values of 0.04, 0.18, and 0.11 μM, respectively [86] (Table 2).

Radicicol (19) was obtained from *Chaetomium c.* associated with *Ephedra fasciculate* and it is a HSP90 (heat shock protein) inhibitor, which is frequently expressed highly in cancer cells. It also showed cytotoxicity to the MCF-7 (breast cancer) cell line at an IC50 value 0.03 μM [55].

Photinides A–F (20a–20f) were obtained from the endophyte *Pestalotiopsis p*. associated with *Roystonea regia*, and all of these γ-lactones at 10 μg/mL exerted cytotoxicity against the MDA-MB-231 (breast cancer) cell line with inhibitory rates of 24.4, 24.2, 23.1, 24.4, and 24.6%, respectively [123] (Table 2).

Eutypellin A (21), isolated from the endophyte *Eutypella* spp. BCC 13199 associated with *Etlingera littoralis*, showed cytotoxicity to KB, MCF-7NCI-H187 (human small-cell lung cancer cells), and nonmalignant Vero cells with IC50 values of 38, 84, 12, and 88 μM, respectively [70].

#### 2.2.10. Lignans

Podophyllotoxin (22), a precursor to the topoisomerase-I-inhibiting anticancer drugs teniposide (23), etoposide (24), and etoposide phosphate, were isolated from the endophyte *Phialocephala f.* associated with *Podophyllum peltatum* [12]. This was also obtained from the endophyte *Trametes h*. associated with *Podophyllum hexandrum* and from the endophyte *Fusarium s*. associated with *Podophyllum hexandrum* [1,79,148] (Table 2).

#### 2.2.11. Peptides

Leucinostatin A was isolated from the endophyte *Acremonium* spp. associated with *Taxus baccata* and was shown to be effective against BT-20 (breast cancer) cell line with an LD50 value of 2 nM [14]. It inhibits the growth of prostate cancer cells through the suppression of IGF-I (Insulin-Like Growth Factor-I) expression in PrSC (prostate stromal cells) [228] (Table 2).

#### 2.2.12. Polyketides

Two novel oblongolides, Y (26a) and Z (26b) (Figure 3a), are produced by the endophyte *Phomopsis* spp. BCC 9789 housed in *Musa acuminate* (a wild banana). Oblongolide Y exhibited cytotoxicity against BC (human breast cancer) cell line (IC50 48 μM) and Oblongolide Z showed cytotoxicity against BC (human breast cancer), KB (human oral epidermoid cancer), NCI-H187 (small-cell lung cancer), and nonmalignant (Vero) cell lines with IC50 values of 26 μM, 37 μM, 32 μM, and 60 μM, respectively [130] (Table 2).

Five tricyclic lactone polyketides, alternariol (27a), alternariol 5-*O*-sulfate (27b), alternariol 5-*O*-methyl ether (27c), altenusin (28a), and desmethylaltenusin (28b) (Figure 3b), were isolated from the endophyte *Alternaria* spp. housed in the leaves of *Polygonum senegalense*. All these compounds manifested significant cytotoxicity against L5178Y (mouse lymphoma cells) with EC50 values of 1.7, 4.5, 7.8, 6.8, and 6.2 μg/mL, respectively [16]. According to another study conducted by Devari et al. in 2014, alternariol 5-*O*-methyl ether showed antiproliferative activity against HL-60 (human promyelocytic leukemia), A549 (lung cancer), PC-3 (prostate cancer), HeLa (cervical cancer), A431 (skin carcinoma), MiaPaka-2 (pancreatic cancer), and T47D (breast cancer) cell lines. Among all these cell lines, HL-60 (human promyelocytic leukemia) cells were most sensitive (IC50 85 μM) to alternariol 5-*O*-methyl ether [25].

Two novel polyketides, leptosphaerone C (29) and penicillenone (30), are produced by an endophytic fungus *Penicillium* spp. JP-1, isolated from *Aegiceras corniculatum*. Leptosphaerone C showed cytotoxicity to A549 (lung carcinoma epithelial) with an IC50 value of 1.45 μM, and penicillenone exhibited activity against P388 (leukemia cells) with an IC50 value of 1.38 μM [103].

Bikaverin (31) was isolated from an endophytic fungus *Fusarium o*. strain CECIS associated with *Cylindropuntia echinocarpa* [77]. It exerted cytotoxic activities against cancer cell lines, MIA PaCa-2 (pancreatic carcinoma), NCI-H460 (non-small cell lung cancer), MCF-7 (human breast cancer), and SF-268 (human CNS cancer) with IC50 values of 0.26, 0.43, 0.42, and 0.38 μM, respectively, showing selective cytotoxicity toward MIA PaCa-2 and NCI-H460. Bikaverin was also proven to be cytotoxic against EAC (Erlich ascites carcinoma), leukemia L5178, and sarcoma 37 cell lines affecting precursor utilization of nucleic acid and protein synthesis [78].

Sequoiatone A (32a) and B (32b), two novel polyketides (Figure 3b), were isolated from a *Sequoia sempervirens* bark endophyte, *Aspergillus p*. These polyketide compounds were tested against 60 diverse human tumor cell lines, and among them, breast cancer cell lines showed the greatest sensitivity [37] (Table 2).

#### 2.2.13. Quinones

Torreyanic acid (33) (Figure 3b), a dimeric quinine, was isolated from an endophyte of *Torreya taxifolia*, *Pestalotiopsis m*. It causes cytotoxicity by apoptosis against A549 (lung carcinoma epithelial) and NEC (human colorectal neuroendocrine cell carcinoma) cell lines with IC50 values of 3.5 μg/mL and 45 μg/mL, respectively [119] (Table 2).

Four endophytes, *Alternaria* spp., *Alternaria a*., *Aspergillus n.*, and *Penicillium* spp., associated with *Tabebuia argentea*, produced the antitumor and anti-metastatic agent lapachol (34) [17,20,21,22]. It acts by interfering with the bioactivities of the topoisomerase enzymes, which are crucial for DNA replication [22]. β-Lapachone showed activity on DU145 (human prostate carcinoma) and MCF-7 (breast cancer cell line) cell lines [20,22]. Additionally, its antitumor and anti-metastatic activities were evident in HepG2 (human hepatocellular liver carcinoma) and Hep3B (human hepatoma cell line) cell lines [19]. Notably, *Aspergillus n*. can be used to produce lapachol in a large scale within a short time [18].

Two bianthraquinone derivatives, Alterporriol K (35a) and L (35b), are produced by the endophytic fungus *Alternaria* spp. ZJ9-6B associated with the mangrove *Aegiceras corniculatum*. Alterporriol K and L exerted moderate cytotoxicity against MDA-MB-435 and MCF-7 (breast cancer cell line) cell lines with IC50 values between 13.1 and 29.1 μM [24].

Cercosporin (36) was isolated from the endophytic fungus *Mycosphaerella* spp., associated with *Psychotria horizontalis*, and exhibited cytotoxicity against MCF-7 [91].

Another endophytic fungus, isolated from the *Salvia officinalis* stem, was *Chaetomium* spp., which produced the cytotoxically active agents, cochliodinol (37) and isocochliodinol (38) (Figure 3b). These compounds were tested against the L5178Y (mouse lymphoma cells) cell line where cochliodinol showed higher cytotoxicity (EC50 7.0 µg/mL) than isocochliodinol (EC50 71.5 µg/mL) [51] (Table 2).

Azaanthraquinones, 7-desmethylscorpinone (39), and 7-desmethyl-6-methylbostrycoidin (40) (Figure 3b) isolated form *Fusarium s*. showed cytotoxic activity against four human tumor cell lines, MDA MB 231, MIA PaCa2, HeLa, and NCI H1975 [229].

#### 2.2.14. Spirobisnaphthalenes

*Mycelia s*., an endophytic fungus isolated from the leaves of *Knightia excelsa*, was shown to produce Spiromamakone A (41) (Figure 3b) that exhibited cytotoxicity to P388 (murine leukemia cell line) at an IC50 value 0.33 μM [90] (Table 2).

A novel spirobisnaphthalene, spiropreussione A (42), was isolated from the endophyte *Preussia* spp. associated with *Aquilaria sinensis*. It displayed cytotoxicity to A2780 (human ovarian carcinoma) and BEL-7404 (human liver carcinoma) cell lines with IC50 values of 2.4 and 3.0 μM, respectively [135].

Diepoxin δ (43), palmarumycin C8 (44), and diepoxins κ and ζ were isolated from the endophytic fungus *Berkleasmium* spp. associated with *Dioscorea zingiberensis*. Diepoxin δ and palmarumycin C8 displayed pronounced cytotoxicity to A-549, A-2780, Bel-7402, BGC-823, and HCT-8 cell lines with IC50 values between 1.28 and 5.83 μM, while diepoxins κ and ζ selectively inhibited A-549 and Bel-7402 cells’ growth showing moderate to weak cytotoxicity [44] (Table 2).

#### 2.2.15. Terpenes (Diterpenes, Sesquiterpenes, Triterpenes)

Several terpenes of plant and fungal origin have been established as potential anticancer drugs (Figure 3b, structures 45–54). Among these, paclitaxel (Taxol) (45) was isolated from *Taxus brevifolia* (Pacific yew tree) [230,231]. However, due to less availability of the pacific yew tree and insignificant yield of this metabolite, scientist have set up other approaches, including tissue culture, chemical synthesis, and semi-synthesis [230,232]. However, this diterpenoid was also reported to be produced by an endophytic fungus, *Taxomyces a*., isolated from the *Taxus brevifolia* [6]. Following this report, a number of paclitaxel producing other endophytes were reported. Some of them are *Bartalinia r*. from the leaves of *Aegle marmelos* [42] and *Pestalotiopsis n*. and *Pestalotiopsis v*. from the plant *Taxus cuspidate* [73]. This metabolite has been found to induce apoptosis when screened against INT-407, BT220, H116, HL251, and HLK210 cell lines [42] (Table 2).

A fusicoccane diterpene, periconicin B (46), was isolated from a *Xylopia aromatica* endophyte, *Periconia a*. It exerted potent cytotoxicity against HeLa (cervical cancer) and CHO (Chinese hamster ovary) cell lines [109].

Four sesquiterpens, trichothecolone (47), 7α-hydroxy-scirpene (48), 8-deoxy-trichothecin (49), and 7α-hydroxytrichodermol (50), were isolated from an endophyte, KLAR 5, housed in the healthy twig of *Knema laurina*. Compounds 47 and 48 were moderately active against BC-1 (human breast cancer cells), KB (Human nasopharyngeal epidermoid tumor), and NCI-H187 (human small-cell lung cancer cells), whereas compounds 49 and 50 showed selective cytotoxic activity against BC-1 and NCI-H187 [86].

Ent-4(15)-eudesmen-11-ol-1-one (51), an eudesmane sesquiterpene, isolated from an *Etlingera littoralis* endophyte, *Eutypella* spp. BCC 13199, showed weak cytotoxicity against KB, MCF7, NCI-H187, and Vero cells with IC50 values of 32, 20, 11, and 32 μM, respectively [70].

Two sesquiterpenes, Merulin A (52a) and Merulin C (52b), are produced by a *Xylocarpus granatum* endophytic fungi, XG8D, where both of them showed significant cytotoxic activity against SW620 (colon cancer) and BT474 (breast cancer) cell lines with IC50 values of 4.84 and 4.11 μg/mL for SW620 and 4.98 and 1.57 μg/mL for BT474, respectively [151].

Three novel eremophilane-type sesquiterpenes (Figure 3b), eremophilanolides 1, 2, and 3 (53a–53c), were isolated from the endophytic fungi *Xylaria* spp. BCC 21097 of the *Licuala spinose* plant and were moderately cytotoxic against KB, MCF-7, and NCI-H187 cell lines [152].

Tauranin (54) is produced by a *Platycladus orientalis* endophyte, *Phyllosticta s*., exhibiting cytotoxicity against MCF-7 (breast cancer), MIA Pa Ca-2 (pancreatic carcinoma), NCI-H460 (non-small cell lung cancer), PC-3 M (metastatic prostate cancer), and SF-268 (CNS cancer- glioma) cell lines with IC50 values of 1.5, 2.8, 4.3, 3.5, and 1.8 μM, respectively [133] (Table 2).

#### 2.2.16. Xanthones

Phomoxanthone A (55a) and B (55b) (Figure 3b), isolated from the endophyte *Phomopsis* spp. BCC 1323 associated with *Tectona grandis*, exerted significant cytotoxicity against KB, BC-1, and nonmalignant Vero cells with IC50 values of 0.99, 0.51, and 1.4 μg/mL, respectively, for phomoxanthone A and 4.1, 0.70, and 1.8 μg/mL, respectively, for phomoxanthone B [129] (Table 2).

### 2.3. Recently Reported Metabolites with Potential Cytotoxicity and the Case of Fusarubin

More than one hundred metabolites have been isolated and evaluated for putative anticancer activities in the years 2018 to 2020. Cytotoxic activities of these endophytic metabolites have been summarized in Table 3. Among the reported metabolites, penicolinate A isolated form *Bionectria* spp. [159] and pyrrocidine A isolated from *Cylindrocarpon* spp. [166] exhibited potent cytotoxicity against against the human ovarian cancer cell line A2780. Fusarithioamide B, a new type benzamide, isolated form *Fusarium c*., showed potent activity against several cell lines [160]. 3-(4-nitrophenyl)-5-phenyl isoxazole was reported to have a potent effect against HepG2 and SMCC-7721 cells [161], while spiciferone F was reported to have a strong effect against MCF7 [162]. Liu et al. isolated two metabolies, namely xylariphthalide A and *cis*-4-hydroxy-6-deoxytalone, and Sharma V. et al. isolated Xylarolide A from *Diaporthe* spp. [163,164]. All these metabolites showed activity towards cancer cells. Three naphthaquinones, anhydrofusarubin, fusarubin, and 3-deoxyfusarubin, and one aza-anthraquinone, bostrycoidin, have potentiality as bioactive compounds against cytotoxicity on vero cells. These metabolites were isolated from a *Fusarium s*. strain isolated from *Casia alata*. [8]. Monolinolein, bafilomycin d, and 3′-hydroxydaidzein displayed a strong effect against A549 cells. These metabolites were isolated from actinomycete strain YBQ59 residing in *Cinnamomum cassia* [167]. *Colletotrichum g*. A12 produced colletotricone A, which showed moderate activity against MCF-7, NCI-H460, HepG-2m and SF-268 tumor cell lines [168]. Mollicellin G, a depsidone, was reported as a moderately active cytotoxic metabolite towards HepG2 and Hela cells [169]. A metabolite of *Pestalotiopsis* spp., named demethylincisterol A3, showed potential cytotoxicity against human cancer cell lines Hela, A549, and HepG [170].

A new type of cytochalasin, named jammosporin A, isolated from endophytic fungi *Rosellinia s*.-*c*., exhibited cytotoxic potential towards MOLT-4 cells [165]. Prenylated diphenyl ethers, namely diorcinol N and analogues isolated from *Arthrinium a*. TE-3, showed moderate cytotoxicity against the human monocytic cell line (THP-1 cell line), with IC50 values of 40.2, 28.3, and 25.9 μM, respectively [233].

An indole diterpenoid, shearilicine, isolated form *Penicillium* spp. (strain ZO-R1-1) of *Zingiber officinale*, showed potent cytotoxicity towards L5178Y cells and A2780 cells [171]. Flavipin from *Chaetomium g*. displayed activity against A549, HT-29, and MCF-7 cells [172]. Emodin, an anthraquinone from *Diaporthe l*., significantly inhibited the growth of murine leukemia P-388 cells [219].

Recently reported metabolites, namely chloroisosulochrin from *Pestalotiopsis t*. (N635) [206], cytosporin W from *Pseudopestalotiopsis t*. [207], terezine E and 14-hydroxyterezine D from *Mucor* spp. [208], citrinin (CIT) and dicitrinin-A from *Penicillium c*. [209], allantopyrone E from *Aspergillus v*. [210], integracin A and B from *Cytospora* spp. [211], (±)-asperteretone F (3a/3b), and compound 6 (name not established in the paper) *Aspergillus t*. [212], sterigmatocystin, a xanthone, from *Paecilamyces* spp. TE-540 [213], mutolide [234] and pramanicin A from *Aplosporella j*. [216], myrothecines H and I from *Paramyrothecium r*. A697 [217], and colletotrichalactone A and colletotrichalactone Ca from *Colletotrichum* spp. JS-0361, exhibited promising activity against different cancer cells [218]. A summary of the putative cytotoxic effects of recently reported endophytic fungal metabolites are summarized in Table 3.

**Figure 4:**
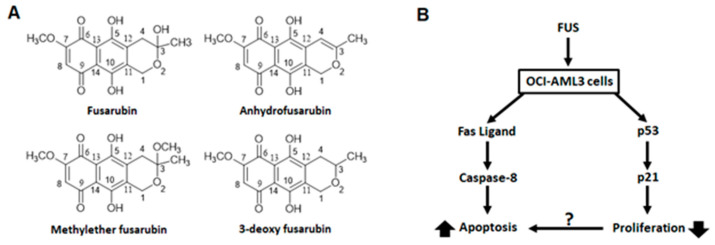
Fusarubin (FUS) and FUS analogues with proposed mechanism of action. (**A**) Structures of FUS derivatives and (**B**) Proposed mechanism of action of FUS on OCI-AML3 cells.

Fusarubin and anhydrofusarubin have been isolated from the endophytic fungi *Cladosporium* residing inside *Rauwolfia* leaves. These compounds inhibited the cell growth of different leukemia cell lines (OCI-AML3, HL-60, U937, and Jurkat) by arresting the cell cycle and augmenting apoptosis. Whereas fusarubin exerted an antiproliferative effect on OCI-AML3 cells by up-regulating p21 in a p53-dependent manner, apoptosis was induced only in a small sub-population of leukemic cells by inducing the production of the Fas ligand (Figure 4) [9].

## 3. Conclusions

Several hundred endophytic fugal metabolites have been isolated to have cytotoxic and antimicrobial effects. Many metabolites are currently available as drugs on the market. Given that plants host endophytes as part of a symbiotic relationship, some plant metabolites might have an endophytic fungal origin. In fact, increasing evidence indicates that some of these plant metabolites are also produced by fungi. Many of the isolated metabolites of endophytic fungi inhabitant medicinal plants have been proved to have cytotoxic effects in vitro. Several of these compounds have been investigated at the molecular level to elucidate the mechanism, since these metabolites are produced in very small quantities by endophytes of plant origin. Due to very insignificant yields and isolation difficulties, these secondary metabolites may not be available to carry out in vivo studies in animal models. Some laboratories applied synthetic approaches to produce natural product derivatives, and one group also tried to synthesize some of these compounds. Optimizing derivatization and synthetic approaches is critical to attain higher yields for animal studies. These approaches will be key for investigating and developing these putative anticancer compounds into treatments.

## Figures and Tables

**Table 1:** Different cell lines against which endophytic fungal derived metabolites showed cytotoxicity.

Cell Lines		Cell Lines	
**A2780S**	Ovarian tumor cell line	**Int-407**	Human intestine cancer
**A2058**	Human melanoma	**Jurkat**	T cell leukemia
**A549**	Lung carcinoma epithelial	**KB**	Human nasopharyngeal epidermoid tumor
**A431**	Skin carcinoma	**K562**	Human leukemia cells
**ACHN**	Renal cells	**L5178Y**	Mouse lymphoma cells
**AsPC-1**	Human pancreatic cancer cells	**MIA Pa Ca-2**	Pancreatic carcinoma
**B16F10**	Skin carcinoma	**MiaPaka-2**	Pancreatic cancer
**BC**	Human breast cancer cell line	**MDA-MB-231**	Breast cancer cell line
**BC-1**	Breast cancer	**MDA-MB-435**	Human breast cancer cell line
**BEL-7402**	Human hepatocellular carcinoma/human hepatoma cell line	**MFC**	Gastric cancer cells in mice
**BEL-7404**	Human hepatocellular carcinoma/human hepatoma cell line	**MCF-7**	Breast cancer cell line
**BGC-823**	Gastric carcinoma	**MOLT-4**	Lymphoblastic leukemia
**BT-220**	Breast cancer cell line	**MRC-5**	Fibroblast-like fetal lung cells
**BT474**	Human breast cancer	**MV4-11**	Human FLT3-ITD mutant AML cell line
**CHO**	Chinese hamster ovary	**NCI-H187**	Human small-cell lung cancer
**DU145**	Human prostate carcinoma	**NCI-H460**	Non-small-cell lung cancer
**EAC**	Ehrlich ascites carcinoma	**NEC**	Colorectal neuroendocrine cell carcinoma
**H116**	Human colon adenocarcinoma	**OVCAR-5**	Human ovarian cancer
**HeLa**	Cervical cancer	**PANC-1**	Human pancreatic carcinoma
**HEp-2**	Human liver cancer	**P388**	Murine leukemia cells
**HepG2**	Human hepatocellular liver carcinoma	**PC-3**	Prostate cancer
**Hep3B**	Human hepatoma cell line	**PC-3 M**	Metastatic prostate cancer
**HM02**	Human gastric carcinoma	**RAW264.7**	Mouse macrophage cell
**HL-60**	Human promyelocytic leukemia cell line	**SF-268**	CNS glioma
**HL251**	Human lung cancer	**SW480**	Human colon cancer cells
**HL-7702**	Normal hepatocyte	**SW-620**	Colon tumor cell line
**HLK 210**	Human leukemia	**SW1116**	Human colon cancer cell line
**HCT-8**	Human colorectal adenocarcinoma	**SW1990**	Human pancreatic cancer cells
**HCT-116**	Colon tumor cell line	**T24**	Bladder carcinoma
**H22**	Hepatic cancer cells in mice	**T47D**	Breast cancer
**H1975**	Non-small-cell lung cancer cells/human lung adenocarcinoma	**THP-1**	Human monocytic cell line
**H522-T1**	Non-small cell lung cancer	**WI-38**	Normal human fibroblast cells
**HT-29**	Human colon cancer line	**U2OS**	Human osteosarcoma cells

**Table 2:** Anticancer compounds from plant-derived endophytic fungi.

Compounds	Chemical Class	Fungal Endophytes	Host Medicinal Plant	Activity Against Cell Lines	IC50 Values	Ref.
Leucinostatin A	Peptide	*Acremonium* spp.	*Taxus baccata* twig	BT-20	2 nM (LD50)	[14]
Allantopyrone A	α-Pyrone	*Allantophomopsis l.* KS-97		A549 cells, HL-60	˃32, 0.32 µM	[15]
Alternariol, Altenusin, Alternariol 5-*O*-sulfate, Alternariol 5-*O*-methyl ether, Desmethylaltenusin	Polyketide	*Alternaria* spp	*Polygonum senegalense* leaves	L5178Y	˂1 × 10^−6^, 1 × 10^−5^, 1 × 10^−5^, 1 × 10^−5^, 1 × 10^−5^ g/mL	[16]
Lapachol	Naphtho-quinone	*Alternaria* spp.	*Tabebuia argentea* leaf	DU145, HepG2, Hep3B & MCF-7 (β-Lapachone)	3.5, 3.5, 3.5 & 5 µM	[17,18,19,20,21,22]
Resveratrodehydes A & B	Stilbenoid (Resveratrol dervatives)	*Alternaria* spp. R6	*Myoporum bontioides* root	MDA-MB-435, HCT-116	˂10 µM	[23]
Alterporriol K, Alterporriol L	Quinones	*Alternaria* spp. ZJ9-6B	*Aegiceras corniculatum*	MDA-MB-435, MCF-7	26.97, 29.11 & 13.11, 20.04 µM	[24]
Alternariol-10-methyl ether	Polyketide	*Alternaria a.*	*Capsicum annum*	HL-60, A549, PC-3, HeLa, A431, MiaPaka-2 and T47D	85, ˃100, ˃100, ˃100, 95, ˃100 and ˃100 µM	[25]
Camptothecine (CPT), 9-methoxy CPT, 10-hydroxy CPT	Alkaloids	*Alternaria a.*	*Miquelia dentata* fruit and seed regions	HCT-116, SW-480, MCF-7	6.59, 7.2, 10.24 µg/mL (crude fungal ethyl acetate extract)	[26]
Chrysin (5,7-dihydroxy flavone)	Flavone	*Alternaria a. (KT380662)*	*Passiflora incarnata* leaves	MCF-7	34.066 µg/mL	[27]
Alternariol 9-methyl ether	Dibenzopyranone	*Alternaria a.*	*Camellia sinensis* branches	U2OS	28.3 µM	[28]
Lapachol	Naphtho-quinone	*Alternaria a.*	*Tabebuia argentea* bark, leaf and stem	DU145, HepG2, Hep3B & MCF-7 (β-Lapachone)	3.5, 3.5, 3.5 & 5 µM	[17,18,19,20,21,22]
(6aR,6bS,7S)-3, 6a, 7,10-tetrahydroxy- 4,9-dioxo-4, 6a, 6b, 7, 8,9-hexahydroperylene	Perylenes	*Alternaria t.*	*Erythrophleum fordii* bark	HCT-8	1.78 µM	[29]
1. Flavasperone, 2. Rubrofusarin B 3. Fonsecinone D	Naphthopyrones	*Aspergillus* sp.	*Limonia acidissima* seeds	1. Hep 3B and U87 MG 2. SW1116 3. SMMC-7721 and A549	1. Between 19.92 and 47.98 µM 2. 4.5 µg/mL 3. ˃10 µg/mL	[30]
Brefeldin A	Lactone	*Aspergillus c.*	*Torreya grandis* bark	HL-60, KB, Hela, MCF-7 and Spc-A-1	1.0–10.0 ng/mL	[31]
9-Deacetoxy fumigaclavine C	Alkaloids	*Aspergillus f.*	*Cynodon dactylon* stem	K562	3.11 µM	[32]
1. Fumitremorgin D, 2. 4,8,10,14-tetramethyl-6-acetoxy-14-[16-acetoxy-19-(20,21- dimethyl)-18-ene]-phenanthrene-1-ene-3,7-dione 3. 12,13-dihydroxy-fumitremorgin C 4. Verruculogen	Alkaloids	*Aspergillus f.*	*Diphylleia sinensis* mainly roots, rhizomes	HepG2	1. 47.5 µM 2. 139.9 µM 3. 4.5 µM 4. 9.8 µM	[33]
2,14-Dihydrox-7-drimen-12,11-olide	Sesquiterpenes	*Aspergillus g.*	*Ipomoea batatas* plant	Hep-G2, MCF-7	61, 41.7 µg/mL	[34]
Nigerapyrones B, D & E Asnipyrones A	Pyrones	*Aspergillus n.* *MA-132*	*Avicennia marina* plant	HepG2, MCF-7, A549, SW1990, MDA-MB-231	86, 105, 43, 38, 48 µM	[35]
Rubrofusarin B	Naphtho-γ-pyrones	*Aspergillus n.*	*Cynodon dactylon*	SW1116	4.5 µg/mL	[36]
Lapachol	Naphtho-quinone	*Aspergillus n.*	*Tabebuia argentea* leaves	DU145, HepG2, Hep3B & MCF-7 (β-Lapachone)	3.5, 3.5, 3.5 & 5 µM	[17,18,19,20,21,22]
1. Sequoiatones A & B 2. Sequoiamonascin A & B	Polyketide	*Aspergillus p.*	*Sequoia sempervirens* inner bark	1. BC 2. MCF7, NCI-H460, SF-268	1. 4 to 10 µM 2. 19 × 10^−4^, 4 × 10^−4^, 15 × 10^−4^ M	[37,38]
Butyrolactone I and Butyrolactone V	Butenolide	*Aspergillus t.*—F7	*Hyptis suaveolens*	MDA-MB-231 and MCF-7	34.4, 17.4 & 22.1, 31.9 µM	[39]
Terrein		*Aspergillus t.* JAS-2	*Achyranthus aspera*	A-549	121.9 µg/mL	[40]
1. Violaceoid A, 2. Violaceoid C, Violaceoid D, 3. Violaceoid F	Hydroquinones	*Aspergillus v.*	Wild Moss (Bryophyta unidentified species)	1. HeLa, MCF-7, Jurkat, MOLT-4, HCT116, RAW264.7 2. Jurkat, MOLT-4 3. HCT116, RAW264.7	1. 24.6, 14.8, 3.1, 3.0, 5.8, 5.6 µM (LD_50_) 2. 8.2, 5.9 & 8.3, 6.2 µM (LD_50_) 3. 6.4, 6.5 µM (LD_50_)	[41]
Taxol	Terpene	*Bartalinia r.*	*Aegle marmelos* leaves	BT 220, H116, Int 407, HL 251 and HLK 210	-	[42]
Depsidone 1	Depsidone	*Pleosporales* (BCC 8616)	unidentified plant leaf of the Hala-Bala forest origin	KB, BC	6.5, 4.1 µg/mL	[43]
1. Diepoxin δ, Palmarumycin C8 2. Diepoxins κ & ζ	Spirobis-naphthalenes	*Berkleasmium* spp.	*Dioscorea zingiberensis*	1. HCT-8, Bel-7402, BGC-823, A 549, A2780 2. Bel-7402 and A 549	1. 1.7, 3.3, 3.3, 3.2, 5.8 & 4.2, 2.5, 2.6, 1.6, 1.3 µM 2. 6.4, 8.7 & 5.1, 8.8 µM	[44]
Verticillin D	Peptide	*Bionectria o.*	*Sonneratia caseolaris* Inner leaf tissues	L5178Y	<0.1 µg/mL (EC50)	[45]
Ophiobolin A	Sesterterpenoid	*Bipolaris s.*	Unidentified	MDA-MB-231	0.4–4.3 µM	[46]
1. Stemphyperylenol 2. Altenuene	1. Polyketide 2. Mycotoxin	*Botryosphaeria d.* KJ-1	*Melia azedarach* stem bark	HCT116	3.13 µM	[47]
Botryorhodine A and B	Depsidone	*Botryosphaeria r.*	*Bidens pilosa* stem	HeLa, K-562	96.97, 36.41 & 0.84, 0.003 µM (CC_50_)	[48]
Cercosporene F	Guanacastane Diterpenes	*Cercospora* spp.	*Fallopia japonica* leaves	HeLa, A549, MCF-7, HCT116 and T24	19.3, 29.7, 46.1, 21.3 & 8.16 µM	[49]
Ceriponol F, Ceriponol G, Ceriponol K	Sesquiterpenes	*Ceriporia l.*	*Huperzia serrata*	HeLa, HepG2, SGC7901	173.2, 32.3, 77.5; 185.1, ˃500.0, ˃500.0 & 47.8, 35.8, 60.2 µM	[50]
Cochliodinol, Isocochliodinol	Quinones	*Chaetomium* spp.	*Salvia* *officinalis Stem*	L5178Y	7.0, 71.5 µg/mL (EC50)	[51]
Chaetocochin C	Diketopiperazine	*Chaetomium* spp.	*Cymbidium goeringii* root	SW-480	0.63 µM	[52]
Chaetocochin G	Indole diketo-piperazines	*Chaetomium* spp. 88194	*Cymbidium goeringii*	MCF-7	8.3 mg/mL	[53]
Chaetominine	Alkaloids	*Chaetomium* spp. IFB-E015	*Adenophora* *axilliflora* leaves	K562, SW1116	21.0, 28.0 nM	[54]
Radicicol	Lactone	*Chaetomium c.*	*Ephedra fasciculate* stem	MCF-7	0.03 µM	[55]
Chaetoglobosin X	Alkaloids	*Chaetomium g.* L18	*Curcuma wenyujin*	H22, MFC	3.125, 6.25 µg/mL	[56]
Chaetoglobosin C, E, F & U, Penochalasin A	Alkaloids	*Chaetomium g.* IFB-E019	*Imperata cylindrica* stem	KB cell line	34.0, 40.0, 48.0 & 16.0, 48.0 µM	[57]
Globosumone A & B	Ester	*Chaetomium g.*	*Ephedra fasciculata*	NCI-H460, MCF-7, SF-268, MIA Pa Ca-2, WI-38	6.50, 21.30, 8.80, 10.60, 13.00 & 24.80, 21.90, 29.10, 30.20, 14.20 µM	[58]
Chaetoglobosins A, F_ex_, F_a_ & 20-dihydrochaetoglobosin	Alkaloids (cytochalasan mycotoxins)	*Chaetomium g.*	*Ginkgo biloba* leaves	HCT116	3.15, 4.43, 5.85, 8.44 µM	[59]
Anhydrofusarubin and methyl ether of Fusarubin	Naphtho-quinones	*Cladosporium* spp.	*Rauwolfia serpentina* leaves	K-562	3.97 & 3.58 µg/mL	[7]
Taxol	Diterpene	*Cladosporium c.*	*Taxus media* inner bark	MCF-7, BT220, H116, INT-407, HL251, HLK210	0.005 to 5 µM	[60,61]
Taxol	Diterpene	*Cladosporium o.*	*Aegle marmelos*, *Coccinia indica and Moringa oleifera*	HCT 15, T47D	3.5, 2.5 µM	[62,63]
Taxol	Diterpene	*Colletotrichum c.*	*Capsicum annuum* fruit	MCF-7, HL 251, HLK 210, BEL7402	0.005 to 5 µM	[64,65]
Tyrosol C	#	*Colletotrichum g.*	*Pandanus amaryllifolius* leaves	A549, HT29, HCT116	-	[66]
Deacetylcytochalasin C and Zygosporin D	Cytochalasins	*Cordyceps t.*	unidentified	95-D	3.67 & 4.04 µM	[67]
1. Cytospolide P, 2. Cytospolide Q	Lactones	*Cytospora* spp.	*Ilex canariensis*	1. A-549, QGY, U973 2. A-549	1. 2.05, 15.82, 28.26 µg/mL 2. 10.55 µg/mL	[68]
Xylarolide	#	*Diaporthe t. GG3F6.*	*Glycyrrhiza glabra* rhizomes	T47D	7 µM	[69]
Taxol	Diterpenes	*Didymostilbe* spp.	*Taxus chinensis var. mairei* old inner bark	MCF-7, HL 251, HLK 210, BEL7402	0.005 to 5 µM	[64,65]
Camptothecin	Alkaloids	*Entrophospora i.*	*Nothapodytes foetida* inner bark	A-549, HEP-2, OVCAR-5	-	[11]
1. Eutypellin A, 2. ent-4(15)-eudesmen-11-ol-1-one	1. γ-Lactone 2. Sesquiterpene	*Eutypella* sp. BCC 13199	*Etlingera littoralis*	NCI-H187, MCF7, KB, Vero cells	1. 12, 84, 38, 88 µM 2. 11, 20, 32, 32 µM	[70]
Camptothecine (CPT), 9-methoxy CPT, 10-hydroxy CPT	Alkaloids	*Fomitopsis* spp.	*Miquelia dentata* fruit and seed regions	HCT-116, SW-480, MCF-7	5.63, 23.5, 10.32 µg/mL (crude fungal ethyl acetate extract)	[26]
Beauvericin	Depsipeptide	*Fusarium o.*	*Cinnamomum kanehirae* bark	PC-3, PANC-1, A549	49.5, 47.2, 10.4 µM	[71]
Taxol	Diterpenes	*Fusarium o.*	*Rhizomphora annamalayana* leaves	BT220, HL251, HLK 210	0.005 to 5 µM	[72,73]
Vincristine	Alkaloids	*Fusarium o.*	*Catharanthus roseus* inner bark	HeLa, MCF7, A549, U251, A431 & HEK293	4.2, 4.5, 5.5, 5.5, 5.8 µg/mL	[74,75]
Beauvericin	Depsipeptide	*Fusarium o.*	*Cinnamomum kanehirae* bark	PC-3, PANC-1, A549	49.5, 47.2, 10.4 µM	[71]
Beauvercin	Depsipeptide	*Fusarium o.*	*Ephedra fasciculata* root	NCI-H460, MIA Pa Ca-2, MCF-7, SF-268, PC-3 M, MDA-MB-231, MRC-5, Hep-G2	1.41, 1.66, 1.81, 2.29, 3.0, 5.0, 4.7–5.0, 8.8–22.2 µM	[76,77]
Beauvercin	Depsipeptide	*Fusarium o.* EPH2RAA	*Cylindropuntia echinocarpus* stem	NCI-H460, MIA Pa Ca-2, MCF-7, SF-268, PC-3 M, MDA-MB-231	1.41, 1.66, 1.81, 2.29, 3.0, 5.0 µM	[77]
Bikaverin	Polyketide	*Fusarium o.* CECIS	*Cylindropuntia echinocarpus* stem	NCI-H460, MIA Pa Ca-2, MCF-7, SF-268, EAC, leukemia L 5178, sarcoma 37	1.41, 1.66, 1.81, 2.29, 0.5, 1.4, 4.2 µg/mL (ED_50_)	[77,78]
Camptothecin (CPT) and 9-methoxy CPT	Alkaloids	*Fusarium s.* (MTCC 9667 and MTCC 9668)	*Apodytes* *dimidiata*	HCT-116, SW-480, MCF-7	7, 8.5, 8 & 7, 8.5, 8 µg/mL	[10,26]
Podophyllotoxin	Lignans	*Fusarium s.*	*Podophyllum hexandrum* roots	#	-	[79]
Camptothecine (CPT), 9-methoxy CPT, 10-hydroxy CPT	Alkaloids	*Fusarium s.*	*Camptotheca acuminata* inner bark	OVCAR-5, HCT-116 SW-480, MCF-7	7, 8.5, 8 & 7, 8.5, 8 µg/mL	[26,80]
Gliocladicillins A & B	Epipolythiodi-oxopiperazines	*Gliocladium* spp. XZC04-CC-302	*Cordyceps sinensis* bark.	HeLa, HepG2, MCF-7	0.50, 0.50,0.20 µg/mL (GI_50_)	[81]
Guignarenone A	Tricyclo-alternarene	*Guignardia b.* PSU-G11	*Garcinia hombroniana* leaves	KB, Vero	0.38, 2.24 µM	[82]
Guignardones Q & S	Meroterpenoids	*Guignardia m.* A348	*Smilax glabra* leaves	MCF-7	83.7 & 92.1 µM	[83]
Cajanol (5-hydroxy-3-(4- hydroxy-2-methoxyphenyl)-7-methoxychroman-4-one)	Flavonoids	*Hypocrea l.*	*Cajanus cajan* roots, stems and leaves	1. A549 2. PC-3, HT-29, HepG2	1. 20.5 µg/mL after 72 h treatment, 24.6 µg/mL after 48 h; and 32.8 µg/mL after 24 h 2. 29.8, 21.4, 33.6 µg/mL (Fungal crude extract)	[84]
Daldinone C & D	Benzo[*j*]fluoranthene	*Hypoxylon t.* IFB-18	*Artemisia annua* surface-sterilized fresh stems	SW1116	49.5 & 41.0 µM	[85]
1. * Brefeldin A, trichothecolone, 7α-hydroxy-scirpene 2. 8-deoxy-trichothecin, 7α-hydroxytrichodermol	* Lactone, Sesquiterpenes (trichothecenes)	KLAR 5 (*Hypocreales*)	*Knema laurina* healthy twig	1. KB, BC-1, NCI-H187 2. BC-1, NCI-H187	1. 0.18, 0.04, 0.1; 12.90, 10.06, 11.31 & ˃75.10, 2.37, 1.73 µM 2. ˃62.81, 0.88, 1.48 & 8.47, 21.53, 27.76 µM	[86]
Taxol	Diterpenes	*Lasiodiplodia t.*	*Morinda citrifolia* leaves	1. MCF-7 2. BT220, H116, INT-407, HL251, HLK210	1. 300 µg/mL 2. 0.005–5.00 µM	[60,87]
Lasiodiplodin	Macrolide	*Lasiodiplodia t.* (MUB-65)	*Myracrodruon urundeuva* branches	HCT-116	11.2 µg/mL	[88]
Vincristine	Alkaloids	*Mycelia s.* 97CY (3)	*Catharanthus roseus* leaves	HeLa, MCF7, U251, A549, A431 & HEK293	4.2, 4.5, 5.5, 5.5, 5.8 µg/mL	[74,89]
Spiromamakone A	Spirobis naphthalene	*Mycelia s.*	*Knightia excelsa* surface-sterilized leaves	P388	0.33 µM	[90]
Cercosporin	Quinones	*Mycosphaerella* spp.	*Psychotria horizontalis*	MCF7	4.68µM	[91]
Arundinone B	Coumarins	*Microsphaeropsis a.*	*Ulmus macrocarpa* stems	T24, A549	35.4, 81.6 µM	[92]
Mycoleptodiscin B	Alkaloids	*Mycoleptodiscus* spp. F0194	*Desmotes incomparabilis* healthy mature leaves	H460, A2058, H522-T1, PC-3, IMR-90	0.66, 0.78, 0.63, 0.60, 0.41 µM	[93]
Myrotheciumone A	Lactone	*Myrothecium r.*	*Ajuga decumbens*	HepG2, SMMC-7721, A549, MCF-7 cells, QSG-7701, HL-7702	5.36, 6.56, 5.88, 7.56, 16.30, 20.69 µM	[94]
Dihydromyrothecine C	Trichothecene Macrolide	*Myrothecium r.* IFB-E012	*Artemisia annua*	KB	44.48 µM	[95]
Camptothecin	Alkaloids	*Neurospora c.*	*Nothapodytes foetida* seed	A-549, HEP-2, OVCAR-5	-	[11,96]
(2R*,4R*)-3,4-dihydro- 4-methoxy-2-methyl-2H-1-benzopyran-5-ol	Pyrans	*Nodulisporium* spp.	*Aquilaria sinensis* stem	SF-268	-	[97]
Brefeldin A	Lactone	*Paecilomyces* spp.	1*. Torreya grandis* 2. *Taxus mairei* bark	HL-60, KB, Hela, MCF-7 and Spc-A-1	10.0, 9.0, 1.8, 2.0 & 1.0 ng/mL	[31]
(22*E*,24*R*)-8,14-epoxyergosta-4,22-diene-3,6- dione	Steroids	*Papulaspora i.*	*Smallanthus sonchifolius* roots & leaves	MDA-MB435, HCT-8, SF295, HL-60	3.3, 14.7, 5.0, 1.6 µM	[98]
1. 19-(α-d-glucopyranosyloxy) isopimara-7,15-dien-3β-ol, 2. 19-(2-acetamido-2- deoxy-α-d glucopyranosyloxy) isopimara- 7,15-dien-3β-ol, 3. 19-(α-d-glucopyranosyloxy) isopimara-7,15-dien-3-one	Diterpenes	*Paraconiothyrium* spp. MY-42	*Fagus* stem	HL60	1. 11.2 µM, 2. 6.7 µM, 3. 9.8 µM	[99]
Brasilamides E	Bisabolane Sesquiterpenoids	*Paraconiothyrium b.* (M3-3341)	*Acer truncatum* branches	MCF-7 and MGC	8.4 & 14.7 µM	[100]
5-Methyl-8-(3-methylbut-2-enyl) furanocoumarin	Coumarins	*Penicillium* spp. ZH16	*Avicennia* sp. leaves	KB, KBV200	5, 10 µg/mL	[101]
1. Penicillenol A_1_, 2. Penicillenol B_1_	Polyketides (tetramic acids derivatives)	*Penicillium* spp. GQ-7	*Aegiceras corniculatum* inner bark	1. A-549, BEL-7402, P388, HL-60 2. HL-60	1. 23.8, 13.03, 8.85, 0.76 µM 2. 3.20 µM	[102]
1. Leptosphaerone C 2. Penicillenone	Polyketides	*Penicillium* spp. JP-1	*Aegiceras corniculatum* inner bark	1. A549 2. P388	1. 1.45 µM 2. 1.38 µM	[103]
Penifupyrone	Funicone	*Penicillium* spp. HSZ-43	*Tripterygium wilfordii* leaves	KB	4.7 µM	[104]
Lapachol	Naphtho-quinone	*Penicillium* spp.	*Tabebuia argentia* leaves	DU145, HepG2, Hep3B & MCF-7 (β-Lapachone)	-	[17,18,19,20,21,22]
Arisugacin B, Arisugacin F	Meroterpenoids	*Penicillium* spp. SXH-65	*Tamarix chinensis* leaves	Hela, HL-60 and K562	59.9, 24.2, 36.2 & 44.4, 45.9, 46.6 µM	[105]
1. *TMC-264*, 2. *PR-toxin*	1. Heptaketide 2. Mycotoxin	*Penicillium ch.* HLit-ROR2	*Hertiera littoralis* root	1,2 >> HuCCA-1, HepG2, A549, MOLT-3, HeLa T47D, MDAMB231, MRC-5, 2. >> HL-60	1,2. 5.62, 3.27, 8.01, 1.36, 4.49, 1.08, 2.81, 12.64 & 0.81, 3.41, 3.59, 0.09, 1.22, 1.00, 2.19, 3.66 µM 2. 0.06 µM	[106]
Citriquinochroman	Alkaloids	*Penicillium ci.*	*Ceratonia siliqua* stem	L5178Y	6.1 µM	[107]
1. (+)-(3S,6S,7 R,8S)- periconone A, 2. (−)-(1R, 4R, 6S, 7S)-2-caren-4,8-olide	Triterpenes	*Periconia* spp.	*Annona muricata* leaves	HCT-8, Bel-7402, BGC-823, A549, A2780, MCF-7	˃10^−5^ M	[108]
Periconicin B	Diterpene	*Periconia a.*	*Xylopia aromatica* leaves	HeLa and CHO	8.0 µM	[109]
Pestalotiopsone F	Chromone	*Pestalotiopsis* spp.	*Rhizophora mucronate* leaves	L5178Y	8.93 µg/mL (EC50)	[110]
Pestalactam A, Pestalactam B	Alkaloids	*Pestalotiopsis* spp.	*Melaleuca quinquenervia* stem	MCF-7, NFF	64.4, 20.2 & 58.5, 12.8 µM	[111]
1. (4*S*,6*S*)-6-[(1*S*,2*R*)-1, 2-dihydroxybutyl]-4-hydroxy-4-methoxytetrahydro-2*H*-pyran-2-one, 2. (6*S*,2*E*)-6-hydroxy-3-methoxy-5-oxodec-2-enoic acid, 3. LL-P880γ 4. LL-P880α 5. Ergosta-5,7,22-trien-3b-ol	Monoterpenoids (1,2)	*Pestalotiopsis* spp. DO14	*Dendrobium officinale*	1–4 >> HL-60 1, 2, 4 and 5 >> LOVO	1–4. 15.24, 30.09, 64.87, 30.75 µM 1,2,4,5. 50.97, 41.91, 68.88 & 65.20 µM	[112]
Siccayne [2-(3-Methyl-3-buten-1-ynyl) Hydroquinone]	Alkyne	*Pestalotiopsis f.*	*Camellia sinensis* branches	HeLa, HT29	48.2, 33.9 µM	[113]
1. Pestalofone F, G & H, Pestalodiol C, 2. Pestaloficiol I, J, K & L	1. Epoxycyclo- hexanediol 2. Isoprenylated chromone	*Pestalotiopsis f.*	*Camellia sinensis* branches	HeLa, MCF-7	1. 14.4, 36.4, 36.4, 16.7 & 11.9, 33.6, 33.6, 57.5 µM 2. ˃136.1, 21.2, 99.3, 8.7 & 136.1, ˃153.8, ˃132.5, 17.4 µM	[114,115]
Pestalrone B	Benzophenones	*Pestalotiopsis k.*	*Camellia sasanqua* stems	HeLa, HepG2, U-251	12.6, 31.7, 5.4 µg/mL	[116]
Taxol	Diterpene	*Pestalotiopsis m.* EF01	*Plectranthus amboinicus* healthy leaves	Hep G2, MCF-7, BT220, HL251	0.5 µM	[117,118]
Torreyanic acid	Quinones	*Pestalotiopsis m.*	*Torreya taxifolia*	NEC, A549	3.5, 45 µg/mL	[119]
Taxol	Diterpene	*Pestalotiopsis m.*	*Taxus wallichiana*	BT220, H116, INT-407, HL251, HLK210, MCF-7	0.005–0.5 µM	[60,120]
Taxol	Diterpenes	*Pestalotiopsis p.* VM1	*Tabebuia pentaphylla*	MCF-7 breast cancer cell line	350 µg/mL	[121]
Photinides A–F, Photipyrone B	γ-Lactones	*Pestalotiopsis p.*	*Roystonea regia*	MDA-MB-231	10 µg/mL (IC25)	[122,123]
Taxol	Diterpenes	*Pestalotiopsis t.*	*Terminalia arjuna* leaves	BT220, H116, INT-407, HL251, HLK210, MCF-7	-	[60,121]
Taxol	Diterpenes	*Pestalotiopsis v.*, *Pestalotiopsis n.*	*Taxus cuspidate* leaves and inner bark	BT220, HL251, HLK 210	-	[73]
Podophyllotoxin	Lignan	*Phialocephala f.*	*Podophyllum peltatum*	Topoisomerase I	-	[12]
Phialomustin A–D	Azaphilone	*Phialophora m.*	*Crocus sativus*	T47D	10, 1, 7, 9.2 µM	[124]
1. 4-hydroxymellein 2. 4,8-dihydroxy-6-methoxy-3-methyl-3,4-dihydro-1H-isochromen-1-one	1. Polyketide 2. Benzopyran	*Phoma* spp.	*Cinnamomum mollissimum*	P388	1. 94.6 (%) 2. 48.8 (%)	[125]
Taxol	Diterpenes	*Phoma b.*	*Ginkgo biloba* leaves	MCF-7, A549, T98G	-	[117]
Camptothecine (CPT) 9-methoxy CPT (9-MeO-CPT), 10-hydroxy CPT (10-OH-CPT)	Alkaloids	*Phomposis* spp.	*Miquelia dentata* fruit and seed regions	HCT-116, SW-480, MCF-7	-	[26]
1. 2-(7′-hydroxyoxooctyl)-3-hydroxy-5-methoxybenzene-acetic acid ethyl ester 2. 3-*O*-(6-*O*-a-L-arabinopyranosyl)- β-d-glucopyranosyl-1,4-dimethoxyxanthone	1. Polyketide 2. Xanthone O-glycoside	*Phomopsis* spp. ZSU-H76	*Excoecaria agallocha* stem	HEp-2 and HepG2	32–64 µg/mL (MIC)	[126,127]
1. Phomopsidone A 2. Diaporthelactone, 7-hydroxy-4,6-dimethyl-3H-isobenzofuran-1-one and 7-methoxy-4,6-dimethyl-3H-isobenzofuran-1-one	1. Depsidone 2. Isobenzo-furanones	*Phomopsis* spp*. A123*	*Kandelia candel* foliage	1. MDA-MB-435 2. Raji cell line	1. 63 µM 2. 27, 47 & 18 µM	[128]
Phomoxanthone A and B	Xanthone	*Phomopsis* spp. BCC 1323	*Tectona grandis*	KB, BC-1, Vero	0.99, 0.51, 1.4 & 4.1, 0.70, 1.8 µg/mL	[129]
1. Oblongolide Y 2. Oblongolide Z	Polyketide (hexaketide *γ*-lactone)	*Phomopsis* spp. BCC 9789	*Musa acuminata* leaf	1. BC 2. KB, BC, NCI-H187, Vero cells	1. 48 µM 2. 37, 26, 32, 60 µM	[130]
18-metoxycytochalasin J, Cytochalasins H and J	Cytochalasins	*Phomopsis* spp.	*Garcinia kola* nut	HeLa	8.18, 35.69 & 3.66 µg/mL (LC50)	[131]
Dicerandrol A, B & C	Ergochromes	*Phomopsis l.*	*Dicerandra frutescens* stem	A549, HCT-116	7, 1.8, 1.8 & 7, 1.8, 7 µg/mL (IC100)	[132]
Tauranin	Sesquiterpene Quinone	*Phyllosticta s.*	*Platycladus orientalis* leaf tissue	NCI-H460, PC-3 M, MCF-7, SF-268, MIA Pa Ca-2	4.3, 3.5, 1.5, 1.8, 2.8 µM	[133]
Ergoflavin	Ergochrome	PM0651480	*Mimusops elengi*	TNF-a, IL-6, ACHN, H460, Panc1, HCT116, and Calu1	1.9, 1.2, 1.2, 4, 2.4, 8, & 1.5 µM	[134]
Spiropreussione A	Spirobis naphthalene	*Preussia* spp.	*Aquilaria sinensis*	A2780, BEL-7404	2.4, 3.0 µM	[135]
Cytochalasin 1, 2, 3 and E	Alkaloids	*Rhinocladiella* spp.	*Tripterygium wilfordii* dead tree limbs	A2780S, HCT-116, SW-620	3.91, 15.6, 3.91; 15.6, 62.5, 15.6; 3.91, -, 15.6 & ˂0.0153, 0.977, 0.244 µg/mL (IC100)	[136]
1. Rhytidones B 2. Rhytidones C, MK3018, Palmarumycin CR1	Spirobis naphthalenes	*Rhytidhysteron* spp.	*Azima sarmentosa* leaves	1. CaSKi 2. MCF-7 and CaSki	1. 22.81 2. 17.30, 20.10, 14.47 & 24.44, 25.59, 21.95 µM	[137]
TMC-264	Heptaketide	*Rhizopycnis v.* Nitaf22	*Nicotiana tabacum*	HCT-116, HepG2, BGC-823, NCIH1650, and A2780	4.2, 5.9, 7.8, 3.2, 3.6 µM	[138]
Rhytidenone H & F	Spirobisnaphthalenes	*Rhytidhysteron r.* AS21B	*Azima sarmentosa*	Ramos and H1975	0.018, 0.252 & 0.048, 1.17 µM	[139]
1. Secalonic acid A, Penicillixanthone A 2. Hypothemycin	1. Tetrahydro-xanthone 2. RAL	*Setophoma t.*	Unidentified (leaf litter collected in a mangrove habitat)	MDA-MB-435 and SW-620	1. 0.16, 0.41 & 0.18, 0.21 µM 2. 0.58, 2.14 µM	[140]
Sphaeropsidin A, Sphaeropsidin D	Diterpenes	*Smardaea* spp. AZ0432	*Ceratodon purpureus* living photosynthetic tissue	MDA-MB-231	1.4, 3.7 µM	[141]
Taxol	Diterpenes	*Stemphylium s.* SBU-16	*Taxus baccata* inner bark	MCF-7, A549, T98G	-	[117,142]
1. Altersolanol A, 2. Alterporriol G and H	Quinones	*Stemphylium g.*	*Mentha pulegium* stem	1. K562, A549, 2. L5178Y	1. ˃1, ˃2 µM 2. 2.7 µg/mL (EC50)	[143,144]
1. 3-Dehydroxymethylbisde-thio-3,10a-bis(methylthio)-gliotoxin 2. Bisdethiobis(methylthio)- Gliotoxin 3. Didehydrobisdethiobis (methylthio)gliotoxin	Alkaloids	*Talaromyces* spp. LGT-2	*Tripterygium wilfordi*	B16	86, 82 & 78% at 500 µg/mL	[145]
Talaperoxide B, Talaperoxide D	Peroxides	*Talaromyces f.*	*Sonneratia apetala* healthy leaves	MCF-7, MDA-MB-435, HepG2, HeLa, PC-3	1.33, 2.78, 1.29, 1.73, 0.89 & 1.92, 0.91, 0.90, 1.31, 0.70 µg/mL	[146]
Vincristine and Vinblastine	Alkaloids	*Talaromyces r.* CrP20	*Catharanthus roseus* leaf tissues	HeLa, MCF7, U251, A549, A431	4.2, 4.5, 5.5, 5.5, 5.8 µg/mL	[74]
Taxol	Terpenes	*Taxomyces a.*	*Taxus brevifolia* inner bark	BT220, H116, INT-407, HL251, MCF-7HLK210	-	[6,60]
Hypericin, Emodin	Polyketides	*Thielavia s.*	*Hypericum perforatum* stem	THP-1	-	[147]
Podophyllotoxin	Lignan	*Trametes h.*	*Podophyllum hexandrum*	Topoisomerase I	-	[148]
Aspochalasin D, Aspochalasin J	Cytochalasan	*Trichoderma g.*	*Panax notoginseng*	HeLa	5.72, 27.4 µM	[149]
Trichothecinol-A	Mycotoxins	*Trichothecium* spp.	*Phyllanthus amarus*	MDA-MBA-231, B16F10	500 µM (LC25), 500 µM (LC50)	[150]
Merulin A Merulin C	Sesquiterpenes	XG8D (a basidiomycete, not better identified)	*Xylocarpus granatum* plant	BT474, SW620	4.98, ˃10 & 4.84, ˃10 µg/mL	[151]
Eremophilanolide 1,2 & 3	Sesquiterpenes	*Xylaria* spp. BCC 21097	*Licuala spinosa*	KB, MCF-7, NCI-H187, Vero cells	3.8–21 µM	[152]
1. 2-Chloro-5-methoxy-3-methylcyclohexa-2,5-diene-1,4-dione 2. Xylariaquinone A	Benzoquinone	*Xylaria* spp.	*Sandoricum koetjape*	Vero cells	1.35, ˃184 µM	[153]
1. Cytochalasin D 2. Cytochalasin C and Q	Cytochalasins	*Xylaria* spp. NC1214	*Hypnum* sp.	1,2 >> NCI-H460, PC-3M, SF-268, MDA-MB-231; 1. >> MCF-7,	D: 1.03, 0.22, 0.43, 1.01 µM; C: 1.65, 1.06, 0.96, 1.72 µM; Q: 1.53, 1.51, 1.31, 1.32; 1.44 µM	[154]
Cytochalasin E	Alkaloids	*Xylaria* spp. *XC-16*	*Toona sinensis*	brine shrimp	2.79 µM (LC50)	[155]
1. Cytochalasin D 2. Ergosterol peroxide	1. Cytochalasins 2. Steroid	*Xylaria* cf. *c.* PK108	Unidentified	1. NCI-H187, KB, Vero cell 2. NCI-H187, Vero cell	1. 5.95, 3.25, 0.36 µg/mL 2. 5.81, 47.95 µg/mL	[156]
Xylariacin A Xylariacin B Xylariacin C	Triterpenes	*Xylarialean* spp. A45	*Annona squamosal* phloem	HepG2	48, 9.7, 46.7% at 20 µg/mL	[157]
Secalonic acid D	Ergochrome	ZSU44 (not better identified)	(unidentified) mangrove plant	HL-60, K562	0.38, 0.43 µM	[158]

* Compounds with IC50 values less than 10 μM are reported.

**Table 3:** Recently (2018–2020) reported potential cytotoxic metabolites isolated from medicinal-plant-associated endophytic fungi.

Sl	Isolated Metabolites *	Fungus Name	Host Medicinal Plant	Reported Activity	References
1	**Penicolinate A**	*Bionectria* spp.	*Raphia taedigera*	Displayed potent cytotoxicity against cells with an IC50 value of 4.1 μM.	[159]
2	**Fusarithioamide B**	*Fusarium c.*	*Anvillea arcinia* (Burm.f.) DC.	Showed selective and potent effect towards BT-549, MCF-7, SKOV-3, and HCT-116 cell lines with IC50s 0.09, 0.21, 1.23, and 0.59 μM, respectively	[160]
3	3-(4-nitrophenyl)-5-phenyl isoxazole	*Aspergillus n.* spp.		Exhibited potent cytotoxic effect on HepG2 and SMCC-7721 cells with the IC50 values were 0.347 and 0.380 mM, respectively	[161]
4	**Spiciferone F**	*Phoma b.*	*Kalidium foliatum* (Pall.) Moq	Exhibited strong biological effect against MCF7 with a half-maximal inhibitory concentration value at 7.73 ± 0.11 μM	[162]
5	**Xylariphthalide A**	*Diaporthe* spp.	*Tylophora ouata*	Displayed cytotoxic activity against human tumor cell lines BGC-823 cells with IC50 values of 1.5 μmol·L^−^¹	[163]
6	***Cis*** **-4-hydroxy-6-deoxytalone**	*Diaporthe* spp.	*Tylophora ouata*	Displayed cytotoxic activity against human tumor cell lines BGC-823 cells with IC50 8.6 μmol·L^−^¹	[163]
7	Xylarolide A	*Diaporthe* spp.	*Datura inoxia*	Showed promisingly inhibited growth of MIAPaCa-2 and PC-3 cells with an IC50 values of 20 14 µM	[164]
8	Jammosporin A	*Rosellinia sanctae-cruciana*	*Albizia lebbeck*	Exhibited promising cytotoxic potential against the human leukemia cancer cell line (MOLT-4)	[165]
9	**Pyrrocidine A** **(Pyridone alkaloid)**	*Cylindrocarpon* spp.	*Sapium ellipticum*	Showed potent cytotoxicity against the human ovarian cancer cell line A2780 with an IC50 value of 1.7 μM	[166]
10	Bostrycoidin	*Fusarium s.*	*Cassia alata* Linn. plant	Significant cytotoxicity against vero cell line	[8]
11	Anhydrofusarubin				
12	**1-Monolinolein**	*Streptomyces c.* YBQ59	*Cinnamomum cassia* plant	Exhibited cytotoxicity against human lung adenocarcinoma EGFR-TKI-resistant A549 cells with IC50 values of 3.6 µM	[167]
13	**Bafilomycin D**			Showed activity against EGFR-TKI-resistant A549 cells with IC50 values 6.7 µM	
14	**3′-Hydroxydaidzein**			Showed activity against EGFR-TKI-resistant A549 cells with IC50 values 7.8 µM	
15	Colletotricone A	*Colletotrichum g. A12*	*Aquilaria sinensis*	Inhibited growth of MCF-7, NCI-H460, HepG-2, and SF-268 tumor cells with IC50 values ranging from 15.7 to 46.8 μM	[168]
16	Mollicellin G	*Chaetomium* spp. Eef-10	*Eucalyptus exserta*	Cytotoxic against two human cancer cell lines HepG2 and Hela withIC50 values of 19.64 and 13.97 µg/mL, respectively	[169]
17	**Demethylincisterol A_3_**	*Pestalotiopsis* spp.	*Rhizophora mucronata*	Showed potent activity against the Hela, A549 and HepG, with IC50 values ranging from 0.17 to 14.16 nM	[170]
18	Shearilicine (1), Paspalinine-13-ene (2), 7-Hydroxypaxilline-13-ene (3), Shearinine *O* (6), Shearinine P (7), emindole SB (10), paspaline (18), 7-hydroxy-13-dehydroxypaxilline (19) *	*Penicillium* spp. (strain ZO-R1-1)	*Zingiber officinale*	**1** showed the most pronounced cytotoxicity against L5178Y (IC50 is 3.6 μM) whereas **2**, **3**, **6**, **7** & **19** exhibited cytotoxicity with IC50 values ranging between 5.3 and 8.1 μM. **1**, **6**, **10** and **18** displayed pronounced cytotoxicity with IC50 values ranging between 5.3 and 8.7 μM against A2780	[171]
19	Flavipin	*Chaetomium g.*	*Couroupita guianensis* Aubl. leaves	Exhibited cytotoxicity toward A549, HT-29, and MCF-7 cancer cells with an IC50 concentration of 9.89 µg/mL, 18 µg/mL, and 54 µg/mL, respectively	[172]
20	**Bellidisin D**	*Phoma b.*	*Tricyrtis maculate* leaves	Exhibited significant cytotoxicity against HL-60, A549, SMMC-7721, MCF-7, and SW480 cells with IC50 value ranged from 3.40 to 15.25 μM	[173]
21	**Epicorazine A**	*Epicoccum n.*	*Salix* sp.	Displayed strong to moderate cytotoxic activities against L5178Y, Ramos, and Jurkat J16 cell lines with IC50_s_ ranging from 1.3 to 28 mM	[174]
22	**Cytochalasin E**	*Aspergillus* spp.	*Pinellia ternata* tubers	Exhibited significant cytotoxicity with an IC50 value of 7.8 μM	[175]
23	Asperchalasin A-F (seco-cytochalasins), Asperlactone G-H (asperlactones)			All the compounds showed cytotoxicity against A-549 with IC50 values ranging from 23.3 to 70.2 μM	
24	**Demethylchaetocochin C, dethiotetra(methylthio)chetomin, chaetoperazine A, 4-formyl-*N*-(30-hydroxypyridin-20-yl) benzamide**	*Chaetomium g.* 7951	*Panax notoginseng* root	Showed cytotoxicity against MCF-7, MDA-MB-231, H460, and HCT-8 cell lines with IC50 values ranging from 4.5 to 65 μM	[176]
25	Chetoseminudin F (**1**), **chaetocochin C (6), ergosterol (8), chetomin A (9), chetomin (12)**	*Chaetomium* spp. SYP-F7950	*Panax notoginseng* Stem	**1** displayed more potent cytotoxic activity against MDA-MB-231 cells than paclitaxel with IC50 of 26.49 μM. **6**, **8**, **9** and **12** exhibited strong cytotoxicity with IC50 values ranging between 2.75 and 8.68 μM against A549 and MDA-MB-231	[177]
26	**Ascomylactam A to C (1–3)**	*Didymella* spp. CYSK-4	*Pluchea indica* healthy branch	**1** and **3** exhibited moderate cytotoxic activities against MDA-MB-231, MDA-MB-435, NCI-H460, PC-3 & HCT116 cell lines with IC50 values ranging between 4.2 and 7.8 μM. **2** showed cytotoxicity towards the MDA-MB-231 and HCT116 cells with IC50s of 6.6 and 4.5 μM, respectively	[178]
27	Pleosporalin F	*Pleosporales* spp. F46	*Mahonia fortunei*	Exhibited moderate cytotoxicity towards MDA-MB-231 cell line with an IC50 value of 22.4 ± 1.1 μM.	[179]
28	19,20-epoxycytochalasins C (**1**) and D (**2**), and 18-deoxy-19,20-epoxy-cytochalasin C (**3**)	*Nemania* spp. UM10M	*Torreya taxifolia* leaf	**1** and **3** displayed moderate toxicity against SK-MEL and BT-549 cell lines. **2** showed moderate toxicity against BT-549 and LLC-PK11 cell lines	[180]
29	**Gartryprostatins A to C (1–3)**	*Aspergillus* spp. GZWMJZ-258	*Garcinia multiflora* fruit	**1**–**3** showed selective cytotoxicity against the cell line, MV4–11, with IC50 values of 7.2, 10.0, and 0.22 μM, respectively	[181]
30	**19,20-epoxycytochalasin C**	*Xylaria* cf. *c.*	*Solanum tuberosum* stem tissues	Displayed significant specific cytotoxic activity against HL-60 cells with an IC50 of 1.11 μM.	[182]
31	Sporulosaldein F	*Paraphaeosphaeria* spp. F03	*Paepalanthus planifolius* leaves	Displayed weak cytotoxic activities against MCF-7 and LM3 cells, with IC50 values of 34.4 and 39.2 µM, respectively.	[183]
32	Trichodermic acid	*Penicillium o.*	*Taxus media* roots	Displayed moderate cytotoxicity towards A549, LN229, MGC, LOVO, and MDA231 with IC50 values of 51.45, 23.43, 39.16, 46.97, and 42.85 μg/mL, respectively.	[184]
33	**Stemphyperylenol (5)**, **(17*R*)-4-hydroxy-17-methylincisterol (10)**	***Alternaria a.***	*Psidium littorale* Raddi leaves	5 showed cytotoxicity against MCF-7 and HepG-4 cell lines (IC50 values of 4.2 ± 0.6 and 7.9 ± 0.9 μM, respectively); **10** exhibited cytotoxicity against HepG-4 cell line with an IC50 value of 9.73 ± 1.2 μM.	[185]
34	**Aspergisocoumrins A & B**	*Aspergillus* spp. HN15-5D	*Acanthus ilicifolius* fresh leaves	Exhibited cytotoxicity against MDA-MB-435 cells (IC50 values of 5.08 ± 0.88 and 4.98 ± 0.74 μM, respectively)	[186]
35	Phomoxanthone A (1) and Penialidin A (2)	*Coniochaeta* spp. F-8	*Ageratina adenophora*	**1** showed a stronger cytotoxicity than **2**	[187]
36	**Macrophin**	*Phoma m.*	*Glycyrrhiza glabra* Linn	Exhibited prominent cytotoxic activity against all the cancer-cell lines (MDA-MB-231, T47D, MCF-7, and MIAPaCa-2 with IC50 values of 14.8, 8.12, 13.0, and 0.9 μM, respectively).	[188]
37	Myrothecines D–G (**1**–**4**), 16-hydroxymytoxin B (**5**), and 14′-dehydrovertisporin (**6**)	*Myrothecium r.*, IFB-E008, IFB-E009, and IFB-E012 strains	*Trachelospermum jasminoides*	Showed cytotoxicity against K562 and SW1116 cells (IC50 values ranging between 56 nM and 16 μM).	[189]
38	Giluterrin	*Aspergillus t.* P63	*Axonopus leptostachyus* roots	Exhibited cytotoxicity against 786-0 and PC-3 cell lines (IC50 of 22.93 μM and 48.55 μM, respectively).	[190]
39	2′-aminodechloromaldoxin (**1**) and 2′-aminodechlorogeodoxin (**2**)	*Pestalotiopsis f.*	*Cinnamomum camphora* branches	1 & 2 displayed moderate cytotoxicity against NCI-H460, SF-268, MCF-7 and PC-3cell lines (IC50 values of 18.63, 20.23, 23.53, 20.48 μM and 16.47, 17.57, 20.79, 19.43 μM, respectively).	[191]
40	**Stachybochartins A, B, C, D and G**.	*Stachybotrys c.* PT2–12	*Pinellia ternata*	Showed cytotoxicity against MDA-MB-231 and U-2OS cells (IC50 values ranging between 4.5 to 21.7 μM).	[192]
41	(*S*)-3,6-dihydroxy-8-methoxy-3-methylisochroman-4-one (**1a**), 6-methoxy-3-methylisochromane-3,8-diol (**2**).	*Aspergillus f.*	*Cordyceps sinensis* fruiting body	**1a** & **2** exhibited moderate growth inhibition against MV4–11 (IC50 values of 38.39 μM and 30.00 μM, respectively).	[193]
42	Flavoglaucin	*Aspergillus* spp. AV-2	*Avicennia marina* healthy leaves	Exhibited most potent cytotoxicity against Caco-2 cells (IC50 of 2.87 μM)	[194]
43	**Peniquinone A (1)** & peniquinone B (**2**)	*Penicillium* spp. L129	*Limonium s.*	**1** showed cytotoxicity against the cell lines, MCF-7, U87, and PC3 (IC50 ranging between 9.01 and 14.59 µM); **2** exhibited relatively weak cytotoxicity against the same cells (IC50 ranging between 13.45 and 25.32 µM)	[195]
44	**Pestalolide B (1), pestalotether F (4)**	*Pestalotiopsis* spp.	*Melaleuca alternifolia* leaves	**1** displayed remarkable inhibitory effect against the cell lines, HL60, U87MG, MDA-MB-231, and HEP-3B cells (IC50 ranging from 1.42 to 5.90 μM); **4** exhibited significant inhibitory potency against HL60 (IC50 5.05 μM)	[196]
45	**Emeridone B (2), Emeridone D (4), Emeridone F (6)**	*Emericella* spp. TJ29	*Hypericum perforatum* root	**2**, **4**, and **6** showed cytotoxicity against cell lines, SMMC-7721 & SW-480 (IC50 values ranging between 8.19 and 18.80 μM). Compound **4** also exhibited cytotoxicity against A-549 (IC50 of 11.33 μM)	[197]
46	Lithocarin B & C, Tenellone H	*Diaporthe l.* A740	*Morinda officinalis* twigs	Displayed weak inhibitory activities against SF-268, MCF-7, HepG-2, and A549 cell lines with IC50 values ranging between 30 and 100 μM	[198]
47	**Cytosporaquinone A**–**D, leucomelone**.	*Cytospora* spp. CCTU A309	*Juglans* (Walnut tree)	All Showed significant cytotoxicity against the cell lines, L929 and KB-3-1 (IC50 values ranging from 2.4 to 26 μg/mL)	[199]
48	**Ilanpyrone (1), methyl** **Asterrate (4)**	*Annulohypoxylon i.*	*Cinnamomum* sp.	**1** showed moderate cytotoxicity against MCF-7 cells (IC50 is 4.79 µM). **4** displayed cytotoxicity towards MCF-7, NCI-H460, and SF-268 cells (IC50 values ranging between 5.46 to 8.56 μM)	[200]
49	**Rhinomilisin A (1), Rhinomilisin G (7) and Gliocladic acid (15)**	*Rhinocladiella s.*	*Acrostichum aureum*	**1**, **7** & **15** exhibited cytotoxic activities against L5178Y (IC50 values of 5.0, 8.7, and 24.4 μM, respectively).	[201]
50	Koninginol B (**2**), 1*R*,3*S*,6*S*,7*R*,10*S*-7-isopropyl-4,10-dimethylbicyclo[4.4.0]dec-4-en-3,10-diol (15), 1*R*,3*R*,6*S*,7*R*,10*S*-7-isopropyl-4,10-dimethylbicyclo[4.4.0]dec-4-en-3,10-diol (**16**)	*Trichoderma k.* A729	*Morinda officinalis* branches	**2**, **15**, and **16** showed antiproliferative activities against A549 (IC50 values of 46.6, 31.3, and 22.2 μM, respectively)	[202]
51	Cytochalasin D1 (1) and C1 (2)	*Xylaria* cf. *cu.*	*Solanum tuberosum* stem tissues	**1** and **2** showed moderate cytotoxicity against HL-60 (IC50 value of 12.7 and 22.3 μM, respectively)	[203]
52	**Bipolahydroquinone C (3), cochlioquinone I (4), cochlioquinones K-M (6–8)**	*Bipolaris* spp. L1–2	*Lycium barbarum* fresh leaves	**3**, **4**, and **6**–**8** exhibited cytotoxic activities against NCIH226 and/or MDA-MB-231 (IC50 values ranging between 5.5 to 9.5 μM)	[204]
53	**Botryosulfuranol A**	*Botryosphaeria m.* strain E224	*Bixa orellana* fresh leaves	Exhibited cytotoxicity against HT-29, HepG2, Caco-2, HeLa, IEC6, and vero cells (IC50 values ranging between 8 to 23.5 μM)	[205]
54	Chloroisosulochrin	*Pestalotiopsis t.* (N635)	*Camellia sinensis* (Theaceae)	Exhibited moderate cytotoxicity towards the HeLa cell line with an IC50 value of 35.2 μM	[206]
55	Pestalotether D			Exerted cytotoxicity against HeLa and MCF-7 cell lines with IC50 values of 60.8 and 22.6 M, respectively	
56	Cytosporins W *	*Pseudopestalotiopsis t.*	*Rhizophora racemosa* mangrove plants	Exhibited potent cytotoxicity towards mouse lymphoma cell line L5178Y with an IC50 value of 3.0 μM	[207]
57	Terezine E and 14-hydroxyterezine D	*Mucor* spp.	*Centaurea stoebe*	Showed potent activity against K-562 and HUVEC cell lines	[208]
58	Citrinin (CIT) and dicitrinin-A	*Penicillium ci.*	*Dichotomaria marginata*	Showed toxicity in *A. saline*, with LC_50_ (24 h) 1.71 μg/mL and 2.29 μg/mL, and LC_50_ (48 h) of 0.54 μg/mL and 0.54 μg/mL, respectively	[209]
59	Allantopyrone E	*Aspergillus v.*	*Avicennia marina* mangrove	Showed cytotoxic effect on HeLa cells with IC50 = 50.97 μM	[210]
60	**Integracin A and B**	*Cytospora* spp.	*Ceriops tagal* (Chinese mangrove)	Both compounds showed promising cytotoxicity towards HepG2 Cells with IC50 values of 5.98 ± 0.12 µM and 9.97 ± 0.06 µM, respectively	[211]
61	**(±)-Asperteretone F (3a/3b)**	*Aspergillus t.*	*Hypericum perforatum*	Potent cytotoxic activities against human pancreatic cancer cells, including AsPC-1, SW1990 and PANC-1 cells, with IC50 values ranging from 1.2 to 15.6 μM	[212]
62	**Sterigmatocystin**	*Paecilamyces* spp. TE-540	*Nicotiana tabacum* L.	showed moderate to strong cytotoxicity towards A549, BT-549, HepG2, and MCF-7 cells with IC50 values ranging from 5.6 to 14.2 µM	[213]
63	Methyl 3-chloroasterric acid	*Pleosporales* spp. SK7.	*Kandelia candel* leaves	Exhibited cytotoxicity against MDA-MB-435 cell with an IC50 of 25.96 ± 0.32 μM	[214]
64	Rhizoperemophilane N	*Rhizopycnis v.*	*Nicotiana tabacum*	Exhibited selective cytotoxicity against NCI-H1650 and BGC823 tumor cells	[215]
65	**Pramanicin A**	*Aplosporella j.*	*Orychophragmus violaceus* (L.) O. E. Schul	exhibited strong cytotoxic activities against human lymphoma (Ramos) and leukemia (Jurkat J16) cells with IC50 values of 4.7 and 4.4 μM, respectively	[216]
66	**Myrothecines H and I**	*Paramyrothecium r.*	*Morinda officinalis*	Both the compounds exhibited promising cytotoxicity against SF-268, NCI-H460, and HepG-2 tumor cell lines with the IC50 ranging from 0.0002–16.2 μM and induced apoptosis of HepG-2 cells	[217]
67	Colletotrichalactone A and colletotrichalactone Ca	*Colletotrichum* spp. JS-0361	*Morus alba*	Exhibited moderate-to-potent cytotoxic activities against MCF7 cells with IC50s of 35.06 and 25.20 µM, respectively	[218]
68	Emodin, (an anthraquinone)	*Diaporthe l.*	*Artocarpus heterophyllus*	exhibited cytotoxicity against murine leukemia P-388 cells with an IC50 value of 0.41 μg/mL	[219]
69	**Demethyli cisterol A_3_**	*Aspergillus t.* YP-2.	*Taxus yunnanensis* bark	Showed cytotoxicity against the A549 and HepG2 cell with IC50 values of 5.34 and 12.03 μM, respectively	[220]
70	Demethylincisterol A_5_			Showed cytotoxicity against the A549 and HepG2 cell with IC50 values of 11.05 and 19.15 μM, respectively	

* Compounds with IC50 values less than 10 μM are reported in bold.

## Data Availability

Not applicable.

## Abbreviations

**Fungus Name**	**Abbreviation**
*Allantophomopsis lycopodina*	*Allantophomopsis l.*
*Alternaria alternata*	*Alternaria a.*
*Alternaria tenuissima*	*Alternaria t.*
*Aspergillus clavatus*	*Aspergillus c.*
*Aspergillus fumigatus*	*Aspergillus f.*
*Aspergillus glaucus*	*Aspergillus g.*
*Aspergillus niger*	*Aspergillus n.*
*Aspergillus parasiticus*	*Aspergillus p.*
*Aspergillus terreus*	*Aspergillus t.*
*Aspergillus violaceofuscus*	*Aspergillus v.*
*Bartalinia robillardoides*	*Bartalinia r.*
*Bionectria ochroleuca*	*Bionectria o.*
*Bipolaris setariae*	*Bipolaris s.*
*Botryosphaeria dothidea*	*Botryosphaeria d.*
*Botryosphaeria rhodina*	*Botryosphaeria r.*
*Ceriporia lacerate*	*Ceriporia l.*
*Chaetomium chiversii*	*Chaetomium c.*
*Chaetomium globosum*	*Chaetomium g.*
*Cladosporium cladosporioides*	*Cladosporium c.*
*Cladosporium oxysporum*	*Cladosporium o.*
*Colletotrichum capsici*	*Colletotrichum c.*
*Colletotrichum gloeosporioides*	*Colletotrichum g.*
*Cordyceps taii*	*Cordyceps t.*
*Diaporthe terebinthifolii*	*Diaporthe t.*
*Entrophospora infrequens*	*Entrophospora i.*
*Fusarium oxysporum*	*Fusarium o.*
*Fusarium solani*	*Fusarium s.*
*Guignardia bidwellii*	*Guignardia b.*
*Guignardia mangiferae*	*Guignardia m.*
*Hypocrea lixii*	*Hypocrea l.*
*Hypoxylon truncatum*	*Hypoxylon t.*
*Lasiodiplodia theobromae*	*Lasiodiplodia t.*
*Mycelia sterilia*	*Mycelia s.*
*Microsphaeropsis arundinis*	*Microsphaeropsis a.*
*Myrothecium roridum*	*Myrothecium r.*
*Neurospora crassa*	*Neurospora c.*
*Papulaspora immersa*	*Papulaspora i.*
*Paraconiothyrium brasiliense*	*Paraconiothyrium b.*
*Penicillium chermesinum*	*Penicillium ch.*
*Penicillium citrinum*	*Penicillium ci.*
*Periconia atropurpurea*	*Periconia a.*
*Pestalotiopsis fici*	*Pestalotiopsis f.*
*Pestalotiopsis karstenii*	*Pestalotiopsis k.*
*Pestalotiopsis microspora*	*Pestalotiopsis m.*
*Pestalotiopsis pauciseta*	*Pestalotiopsis pa.*
*Pestalotiopsis photiniae*	*Pestalotiopsis ph.*
*Pestalotiopsis terminaliae*	*Pestalotiopsis t.*
*Pestalotiopsis versicolor*	*Pestalotiopsis v.*
*Pestalotiopsis neglecta*	*Pestalotiopsis n.*
*Phialocephala fortinii*	*Phialocephala f.*
*Phialophora mustea*	*Phialophora m.*
*Phoma betae*	*Phoma b.*
*Phomopsis longicolla*	*Phomopsis l.*
*Phyllosticta spinarum*	*Phyllosticta s.*
*Rhizopycnis vagum*	*Rhizopycnis v.*
*Rhytidhysteron rufulum*	*Rhytidhysteron r.*
*Setophoma terrestris*	*Setophoma t.*
*Stemphylium sedicola*	*Stemphylium s.*
*Stemphylium globuliferum*	*Stemphylium g.*
*Talaromyces flavus*	*Talaromyces f.*
*Talaromyces radicus*	*Talaromyces r.*
*Taxomyces andreanae*	*Taxomyces a.*
*Thielavia subthermophila*	*Thielavia s.*
*Trametes hirsuta*	*Trametes h.*
*Trichoderma gamsii*	*Trichoderma g.*
*Xylaria* cf. *cubensis*	*Xylaria* cf. *c.*

## References

[1] Kumar V., Rai S., Gaur P., Fatima T., Verma V.C., Gange A.C. (2014). Endophytic Fungi: Novel Sources of Anticancer Molecules. Advances in Endophytic Research.

[2] Gunatilaka A.A.L. (2006). Natural Products from Plant-Associated Microorganisms: Distribution, Structural Diversity, Bioactivity, and Implications of Their Occurrence. J. Nat. Prod..

[3] Zhang H.W., Song Y.C., Tan R.X. (2006). Biology and Chemistry of Endophytes. Nat. Prod. Rep..

[4] Aly A.H., Debbab A., Kjer J., Proksch P. (2010). Fungal Endophytes from Higher Plants: A Prolific Source of Phytochemicals and Other Bioactive Natural Products. Fungal Divers..

[5] Staniek A., Woerdenbag H.J., Kayser O. (2008). Endophytes: Exploiting Biodiversity for the Improvement of Natural Product-Based Drug Discovery. J. Plant Interact..

[6] Stierle A., Strobel G., Stierle D. (1993). Taxol and Taxane Production by *Taxomyces Andreanae*, an Endophytic Fungus of Pacific Yew. Sci.-N. Y. THEN Wash..

[7] Khan M.I.H., Sohrab M.H., Rony S.R., Tareq F.S., Hasan C.M., Mazid M.A. (2016). Cytotoxic and Antibacterial Naphthoquinones from an Endophytic Fungus, *Cladosporium* sp.. Toxicol. Rep..

[8] Khan N., Afroz F., Begum N., Roy Rony S., Sharmin S., Moni F., Mahmood Hasan C., Shaha K., Sohrab H. (2018). Endophytic *Fusarium Solani*: A Rich Source of Cytotoxic and Antimicrobial Napthaquinone and Aza-Anthraquinone Derivatives. Toxicol. Rep..

[9] Adorisio S., Fierabracci A., Muscari I., Liberati A.M., Cannarile L., Thuy T.T., Sung T.V., Sohrab H., Hasan C.M., Ayroldi E. (2019). Fusarubin and Anhydrofusarubin Isolated from a Cladosporium Species Inhibit Cell Growth in Human Cancer Cell Lines. Toxins.

[10] Shweta S., Zuehlke S., Ramesha B.T., Priti V., Mohana Kumar P., Ravikanth G., Spiteller M., Vasudeva R., Uma Shaanker R. (2010). Endophytic Fungal Strains of *Fusarium Solani*, from Apodytes Dimidiata E. Mey. Ex Arn (Icacinaceae) Produce Camptothecin, 10-Hydroxycamptothecin and 9-Methoxycamptothecin. Phytochemistry.

[11] Puri S.C., Verma V., Amna T., Qazi G.N., Spiteller M. (2005). An Endophytic Fungus from Nothapodytes Foetida That Produces Camptothecin. J. Nat. Prod..

[12] Eyberger A.L., Dondapati R., Porter J.R. (2006). Endophyte Fungal Isolates from Podophyllum Peltatum Produce Podophyllotoxin. J. Nat. Prod..

[13] Kusari S., Lamshöft M., Spiteller M. (2009). *Aspergillus Fumigatus* Fresenius, an Endophytic Fungus from *Juniperus Communis* L. Horstmann as a Novel Source of the Anticancer pro-Drug Deoxypodophyllotoxin. J. Appl. Microbiol..

[14] Strobel G.A., Hess W.M. (1997). Glucosylation of the Peptide Leucinostatin A, Produced by an Endophytic Fungus of European Yew, May Protect the Host from Leucinostatin Toxicity. Chem. Biol..

[15] Yokoigawa J., Morimoto K., Shiono Y., Uesugi S., Kimura K., Kataoka T. (2015). Allantopyrone A, an α-Pyrone Metabolite from an Endophytic Fungus, Inhibits the Tumor Necrosis Factor α-Induced Nuclear Factor ΚB Signaling Pathway. J. Antibiot..

[16] Aly A.H., Edrada-Ebel R., Indriani I.D., Wray V., Müller W.E., Totzke F., Zirrgiebel U., Schächtele C., Kubbutat M.H., Lin W.H. (2008). Cytotoxic Metabolites from the Fungal Endophyte *Alternaria* sp. and Their Subsequent Detection in Its Host Plant Polygonum Senegalense. J. Nat. Prod..

[17] Balassiano I.T., De Paulo S.A., Henriques Silva N., Cabral M.C., da Gloria da Costa Carvalho M. (2005). Demonstration of the Lapachol as a Potential Drug for Reducing Cancer Metastasis. Oncol. Rep..

[18] Govindappa M. (2014). First Report of Anticancer Agent, Lapachol Producing Endophyte, *Aspergillus Niger* of Tabebuia Argentea and Its in Vitro Cytotoxicity Assays. Bangladesh J. Pharmacol..

[19] KIM S.O., KWON J.I., JEONG Y.K., KIM G.Y., KIM N.D., CHOI Y.H. (2007). Induction of Egr-1 Is Associated with Anti-Metastatic and Anti-Invasive Ability of β-Lapachone in Human Hepatocarcinoma Cells. Biosci. Biotechnol. Biochem..

[20] Lee J.H., Cheong J., Park Y.M., Choi Y.H. (2005). Down-Regulation of Cyclooxygenase-2 and Telomerase Activity by β-Lapachone in Human Prostate Carcinoma Cells. Pharmacol. Res..

[21] Sadananda T.S., Nirupama R., Chaithra K., Govindappa M., Chandrappa C.P., Vinay Raghavendra B. (2011). Antimicrobial and Antioxidant Activities of Endophytes from Tabebuia Argentea and Identification of Anticancer Agent (Lapachol). J. Med. Plants Res..

[22] Wuerzberger S.M., Pink J.J., Planchon S.M., Byers K.L., Bornmann W.G., Boothman D.A. (1998). Induction of Apoptosis in MCF-7:WS8 Breast Cancer Cells by β-Lapachone. Cancer Res..

[23] Wang J., Cox D.G., Ding W., Huang G., Lin Y., Li C. (2014). Three New Resveratrol Derivatives from the Mangrove Endophytic Fungus *Alternaria* sp.. Mar. Drugs.

[24] Huang C.-H., Pan J.-H., Chen B., Yu M., Huang H.-B., Zhu X., Lu Y.-J., She Z.-G., Lin Y.-C. (2011). Three Bianthraquinone Derivatives from the Mangrove Endophytic Fungus *Alternaria* sp. ZJ9-6B from the South China Sea. Mar. Drugs.

[25] Devari S., Jaglan S., Kumar M., Deshidi R., Guru S., Bhushan S., Kushwaha M., Gupta A.P., Gandhi S.G., Sharma J.P. (2014). Capsaicin Production by *Alternaria Alternata*, an Endophytic Fungus from Capsicum Annum; LC–ESI–MS/MS Analysis. Phytochemistry.

[26] Shweta S., Gurumurthy B.R., Ravikanth G., Ramanan U.S., Shivanna M.B. (2013). Endophytic Fungi from Miquelia Dentata Bedd., Produce the Anti-Cancer Alkaloid, Camptothecine. Phytomed. Int. J. Phytother. Phytopharm..

[27] Seetharaman P., Gnanasekar S., Chandrasekaran R., Chandrakasan G., Kadarkarai M., Sivaperumal S. (2017). Isolation and Characterization of Anticancer Flavone Chrysin (5,7-Dihydroxy Flavone)-Producing Endophytic Fungi from *Passiflora Incarnata* L. Leaves. Ann. Microbiol..

[28] Wang Y., Yang M.-H., Wang X.-B., Li T.-X., Kong L.-Y. (2014). Bioactive Metabolites from the Endophytic Fungus *Alternaria Alternata*. Fitoterapia.

[29] Fang Z.F., Yu S.S., Zhou W.Q., Chen X.G., Ma S.G., Li Y., Qu J. (2012). A New Isocoumarin from Metabolites of the Endophytic Fungus *Alternaria Tenuissima* (Nees & T. Nees: Fr.) Wiltshire. Chin. Chem. Lett..

[30] Siriwardane A.M.D.A., Kumar N.S., Jayasinghe L., Fujimoto Y. (2015). Chemical Investigation of Metabolites Produced by an Endophytic *Aspergillus* sp. Isolated from Limonia Acidissima. Nat. Prod. Res..

[31] Wang J., Huang Y., Fang M., Zhang Y., Zheng Z., Zhao Y., Su W. (2002). Brefeldin A, a Cytotoxin Produced by *Paecilomyces* sp. and *Aspergillus Clavatus* Isolated from Taxus Mairei and Torreya Grandis. FEMS Immunol. Med. Microbiol..

[32] Ge H.M., Yu Z.G., Zhang J., Wu J.H., Tan R.X. (2009). Bioactive Alkaloids from Endophytic *Aspergillus Fumigatus*. J. Nat. Prod..

[33] Liang Z., Zhang T., Zhang X., Zhang J., Zhao C. (2015). An Alkaloid and a Steroid from the Endophytic Fungus *Aspergillus Fumigatus*. Molecules.

[34] Asker M., Mohamed S.F., Mahmoud M.G., Sayed O.H.E. (2013). Antioxidant and Antitumor Activity of a New Sesquiterpene Isolated from Endophytic Fungus *Aspergillus Glaucus*. Int. J. PharmTech Res..

[35] Liu D., Li X.-M., Meng L., Li C.-S., Gao S.-S., Shang Z., Proksch P., Huang C.-G., Wang B.-G. (2011). Nigerapyrones A-H, α-Pyrone Derivatives from the Marine Mangrove-Derived Endophytic Fungus *Aspergillus Niger* MA-132. J. Nat. Prod..

[36] Song Y.C., Li H., Ye Y.H., Shan C.Y., Yang Y.M., Tan R.X. (2004). Endophytic Naphthopyrone Metabolites Are Co-Inhibitors of Xanthine Oxidase, SW1116 Cell and Some Microbial Growths. FEMS Microbiol. Lett..

[37] Stierle A.A., Stierle D.B., Bugni T. (1999). Sequoiatones A and B: Novel Antitumor Metabolites Isolated from a Redwood Endophyte. J. Org. Chem..

[38] Stierle D.B., Stierle A.A., Bugni T. (2003). Sequoiamonascins A–D: Novel Anticancer Metabolites Isolated from a Redwood Endophyte. J. Org. Chem..

[39] Da Silva I.P., Brissow E., Filho L.C.K., Senabio J., de Siqueira K.A., Filho S.V., Damasceno J.L., Mendes S.A., Tavares D.C., Magalhães L.G. (2017). Bioactive Compounds of *Aspergillus Terreus*—F7, an Endophytic Fungus from *Hyptis Suaveolens* (L.) Poit. World J. Microbiol. Biotechnol..

[40] Goutam J., Sharma G., Tiwari V.K., Mishra A., Kharwar R.N., Ramaraj V., Koch B. (2017). Isolation and Characterization of “Terrein” an Antimicrobial and Antitumor Compound from Endophytic Fungus *Aspergillus Terreus* (JAS-2) Associated from Achyranthus Aspera Varanasi, India. Front. Microbiol..

[41] Myobatake Y., Takemoto K., Kamisuki S., Inoue N., Takasaki A., Takeuchi T., Mizushina Y., Sugawara F. (2014). Cytotoxic Alkylated Hydroquinone, Phenol, and Cyclohexenone Derivatives from *Aspergillus Violaceofuscus* Gasperini. J. Nat. Prod..

[42] Gangadevi V., Muthumary J. (2008). Taxol, an Anticancer Drug Produced by an Endophytic Fungus *Bartalinia Robillardoides* Tassi, Isolated from a Medicinal Plant, Aegle Marmelos Correa Ex Roxb. World J. Microbiol. Biotechnol..

[43] Pittayakhajonwut P., Dramae A., Madla S., Lartpornmatulee N., Boonyuen N., Tanticharoen M. (2006). Depsidones from the Endophytic Fungus BCC 8616. J. Nat. Prod..

[44] Shan T., Tian J., Wang X., Mou Y., Mao Z., Lai D., Dai J., Peng Y., Zhou L., Wang M. (2014). Bioactive Spirobisnaphthalenes from the Endophytic Fungus *Berkleasmium* sp.. J. Nat. Prod..

[45] Ebrahim W., Kjer J., El Amrani M., Wray V., Lin W., Ebel R., Lai D., Proksch P. (2012). Pullularins E and F, Two New Peptides from the Endophytic Fungus *Bionectria Ochroleuca* Isolated from the Mangrove Plant Sonneratia Caseolaris. Mar. Drugs.

[46] Bhatia D.R., Dhar P., Mutalik V., Deshmukh S.K., Verekar S.A., Desai D.C., Kshirsagar R., Thiagarajan P., Agarwal V. (2016). Anticancer Activity of Ophiobolin A, Isolated from the Endophytic Fungus *Bipolaris Setariae*. Nat. Prod. Res..

[47] Xiao J., Zhang Q., Gao Y.-Q., Tang J.-J., Zhang A.-L., Gao J.-M. (2014). Secondary Metabolites from the Endophytic *Botryosphaeria Dothidea* of Melia Azedarach and Their Antifungal, Antibacterial, Antioxidant, and Cytotoxic Activities. J. Agric. Food Chem..

[48] Abdou R., Scherlach K., Dahse H.-M., Sattler I., Hertweck C. (2010). Botryorhodines A–D, Antifungal and Cytotoxic Depsidones from *Botryosphaeria Rhodina*, an Endophyte of the Medicinal Plant Bidens Pilosa. Phytochemistry.

[49] Feng Y., Ren F., Niu S., Wang L., Li L., Liu X., Che Y. (2014). Guanacastane Diterpenoids from the Plant Endophytic Fungus *Cercospora* sp.. J. Nat. Prod..

[50] Ying Y.-M., Shan W.-G., Zhang L.-W., Zhan Z.-J. (2013). Ceriponols A-K, Tremulane Sesquitepenes from *Ceriporia Lacerate* HS-ZJUT-C13A, a Fungal Endophyte of Huperzia Serrata. Phytochemistry.

[51] Debbab A., Aly H.A., Edrada-Ebel R.A., Müller W.E., Mosaddak M., Hakiki A., Ebel R., Proksch P. (2009). Bioactive Secondary Metabolites from the Endophytic Fungus *Chaetomium* sp. Isolated from Salvia Officinalis Growing in Morocco. Biotechnol. Agron. Soc. Environ..

[52] Wang F., Jiang J., Hu S., Ma H., Zhu H., Tong Q., Cheng L., Hao X., Zhang G., Zhang Y. (2017). Secondary Metabolites from Endophytic Fungus *Chaetomium* sp. Induce Colon Cancer Cell Apoptotic Death. Fitoterapia.

[53] Wang F., Tong Q., Ma H., Xu H., Hu S., Ma W., Xue Y., Liu J., Wang J., Song H. (2015). Indole Diketopiperazines from Endophytic *Chaetomium* Sp 88194 Induce Breast Cancer Cell Apoptotic Death. Sci. Rep..

[54] Jiao R.H., Xu S., Liu J.Y., Ge H.M., Ding H., Xu C., Zhu H.L., Tan R.X. (2006). Chaetominine, a Cytotoxic Alkaloid Produced by Endophytic *Chaetomium* sp. IFB-E015. Org. Lett..

[55] Turbyville T.J., Wijeratne E.K., Liu M.X., Burns A.M., Seliga C.J., Luevano L.A., David C.L., Faeth S.H., Whitesell L., Gunatilaka A.L. (2006). Search for Hsp90 Inhibitors with Potential Anticancer Activity: Isolation and SAR Studies of Radicicol and Monocillin I from Two Plant-Associated Fungi of the Sonoran Desert. J. Nat. Prod..

[56] Wang Y., Xu L., Ren W., Zhao D., Zhu Y., Wu X. (2012). Bioactive Metabolites from *Chaetomium Globosum* L18, an Endophytic Fungus in the Medicinal Plant Curcuma Wenyujin. Phytomed. Int. J. Phytother. Phytopharm..

[57] Ding G., Song Y.C., Chen J.R., Xu C., Ge H.M., Wang X.T., Tan R.X. (2006). Chaetoglobosin U, a Cytochalasan Alkaloid from Endophytic *Chaetomium Globosum* IFB-E019. J. Nat. Prod..

[58] Bashyal B.P., Wijeratne E.K., Faeth S.H., Gunatilaka A.L. (2005). Globosumones A- C, Cytotoxic Orsellinic Acid Esters from the Sonoran Desert Endophytic Fungus *Chaetomium Globosum* 1. J. Nat. Prod..

[59] Li H., Xiao J., Gao Y.-Q., Tang J., Zhang A.-L., Gao J.-M. (2014). Chaetoglobosins from *Chaetomium Globosum*, an Endophytic Fungus in Ginkgo Biloba, and Their Phytotoxic and Cytotoxic Activities. J. Agric. Food Chem..

[60] Gangadevi V., Muthumary J. (2009). Taxol Production by *Pestalotiopsis Terminaliae*, an Endophytic Fungus of Terminalia Arjuna (Arjun Tree). Biotechnol. Appl. Biochem..

[61] Zhang P., Zhou P.-P., Yu L.-J. (2009). An Endophytic Taxol-Producing Fungus from Taxus Media, *Cladosporium Cladosporioides* MD2. Curr. Microbiol..

[62] Gokul Raj K., Manikandan R., Arulvasu C., Pandi M. (2015). Anti-Proliferative Effect of Fungal Taxol Extracted from *Cladosporium Oxysporum* against Human Pathogenic Bacteria and Human Colon Cancer Cell Line HCT 15. Spectrochim. Acta A Mol. Biomol. Spectrosc..

[63] Raj K.G., Sambantham S., Manikanadan R., Arulvasu C., Pandi M. (2014). Fungal Taxol Extracted from *Cladosporium Oxysporum* Induces Apoptosis in T47D Human Breast Cancer Cell Line. Asian Pac. J. Cancer Prev..

[64] Kumaran R.S., Jung H., Kim H.J. (2011). In Vitro Screening of Taxol, an Anticancer Drug Produced by the Fungus, *Colletotrichum Capsici*. Eng. Life Sci..

[65] Wang Y., Tang K. (2011). A New Endophytic Taxol-and Baccatin III-Producing Fungus Isolated from Taxus Chinensis Var. Mairei. Afr. J. Biotechnol..

[66] Bungihan M., Tan A.M., Takayama H., Cruz D.E., Nonato G.M. (2013). A New Macrolide Isolated from the Endophytic Fungus *Colletotrichum* sp.. Philipp. Sci. Lett..

[67] Li X.-G., Pan W.-D., Lou H.-Y., Liu R.-M., Xiao J.-H., Zhong J.-J. (2015). New Cytochalasins from Medicinal Macrofungus Crodyceps Taii and Their Inhibitory Activities against Human Cancer Cells. Bioorg. Med. Chem. Lett..

[68] Lu S., Sun P., Li T., Kurtán T., Mándi A., Antus S., Krohn K., Draeger S., Schulz B., Yi Y. (2011). Bioactive Nonanolide Derivatives Isolated from the Endophytic Fungus *Cytospora* sp.. J. Org. Chem..

[69] Yedukondalu N., Arora P., Wadhwa B., Malik F.A., Vishwakarma R.A., Gupta V.K., Riyaz-Ul-Hassan S., Ali A. (2017). Diapolic Acid A–B from an Endophytic Fungus, *Diaporthe Terebinthifolii* Depicting Antimicrobial and Cytotoxic Activity. J. Antibiot..

[70] Isaka M., Palasarn S., Lapanun S., Chanthaket R., Boonyuen N., Lumyong S. (2009). γ-Lactones and Ent-Eudesmane Sesquiterpenes from the Endophytic Fungus *Eutypella* sp. BCC 13199. J. Nat. Prod..

[71] Wang Q.-X., Li S.-F., Zhao F., Dai H.-Q., Bao L., Ding R., Gao H., Zhang L.-X., Wen H.-A., Liu H.-W. (2011). Chemical Constituents from Endophytic Fungus *Fusarium Oxysporum*. Fitoterapia.

[72] Elavarasi A., Rathna G.S., Kalaiselvam M. (2012). Taxol Producing Mangrove Endophytic Fungi *Fusarium Oxysporum* from Rhizophora Annamalayana. Asian Pac. J. Trop. Biomed..

[73] Kumaran R.S., Kim H.J., Hur B.-K. (2010). Taxol Promising Fungal Endophyte, Pestalotiopsis Species Isolated from Taxus Cuspidata. J. Biosci. Bioeng..

[74] Palem P.P.C., Kuriakose G.C., Jayabaskaran C. (2015). An Endophytic Fungus, *Talaromyces Radicus*, Isolated from Catharanthus Roseus, Produces Vincristine and Vinblastine, Which Induce Apoptotic Cell Death. PLoS ONE.

[75] Zhang L., Guo B., Li H., Zeng S., Shao H., Gu S., Wei R. (2000). Preliminary Study on the Isolation of Endophytic Fungus of Catharanthus Roseus and Its Fermentation to Produce Products of Therapeutic Value. Chin. Tradit. Herb. Drugs.

[76] Ivanova L., Skjerve E., Eriksen G.S., Uhlig S. (2006). Cytotoxicity of Enniatins A, A1, B, B1, B2 and B3 from Fusarium Avenaceum. Toxicon Off. J. Int. Soc. Toxinology.

[77] Zhan J., Burns A.M., Liu M.X., Faeth S.H., Gunatilaka A.A.L. (2007). Search for Cell Motility and Angiogenesis Inhibitors with Potential Anticancer Activity: Beauvericin and Other Constituents of Two Endophytic Strains of *Fusarium Oxysporum*. J. Nat. Prod..

[78] Fuska J., Proksa B., Fusková A. (1975). New Potential Cytotoxic and Antitumor Substances I. In Vitro Effect of Bikaverin and Its Derivatives on Cells of Certain Tumors. Neoplasma.

[79] Nadeem M., Ram M., Alam P., Ahmad M.M., Mohammad A., Al-Qurainy F., Khan S., Abdin M.Z. (2012). *Fusarium Solani*, P1, a New Endophytic Podophyllotoxin-Producing Fungus from Roots of Podophyllum Hexandrum. Afr. J. Microbiol. Res..

[80] Kusari S., Zühlke S., Spiteller M. (2009). An Endophytic Fungus from *Camptotheca Acuminata* That Produces Camptothecin and Analogues. J. Nat. Prod..

[81] Chen Y., Gou H., Du Z., Liu X.-Z., Che Y., Ye X. (2009). Ecology-Based Screen Identifies New Metabolites from a Cordyceps-Colonizing Fungus as Cancer Cell Proliferation Inhibitors and Apoptosis Inducers. Cell Prolif..

[82] Sommart U., Rukachaisirikul V., Trisuwan K., Tadpetch K., Phongpaichit S., Preedanon S., Sakayaroj J. (2012). Tricycloalternarene Derivatives from the Endophytic Fungus *Guignardia Bidwellii* PSU-G11. Phytochem. Lett..

[83] Sun Z.-H., Liang F.-L., Wu W., Chen Y.-C., Pan Q.-L., Li H.-H., Ye W., Liu H.-X., Li S.-N., Tan G.-H. (2015). Guignardones P–S, New Meroterpenoids from the Endophytic Fungus *Guignardia Mangiferae* A348 Derived from the Medicinal Plant Smilax Glabra. Molecules.

[84] Zhao J., Li C., Wang W., Zhao C., Luo M., Mu F., Fu Y., Zu Y., Yao M. (2013). *Hypocrea Lixii*, Novel Endophytic Fungi Producing Anticancer Agent Cajanol, Isolated from Pigeon Pea (*Cajanus Cajan* [L.] Millsp.). J. Appl. Microbiol..

[85] Gu W., Ge H.M., Song Y.C., Ding H., Zhu H.L., Zhao X.A., Tan R.X. (2007). Cytotoxic Benzo [j] Fluoranthene Metabolites from *Hypoxylon Truncatum* IFB-18, an Endophyte of Artemisia Annua. J. Nat. Prod..

[86] Chinworrungsee M., Wiyakrutta S., Sriubolmas N., Chuailua P., Suksamrarn A. (2008). Cytotoxic Activities of Trichothecenes Isolated from an Endophytic Fungus Belonging to Order Hypocreales. Arch. Pharm. Res..

[87] Pandi M., Kumaran R.S., Choi Y.-K., Kim H.J., Muthumary J. (2011). Isolation and Detection of Taxol, an Anticancer Drug Produced from *Lasiodiplodia Theobromae*, an Endophytic Fungus of the Medicinal Plant Morinda Citrifolia. Afr. J. Biotechnol..

[88] Sobreira A.C.M., Pessoa O.D.L., Florêncio K.G.D., Wilke D.V., Freire F.C.O., Gonçalves F.J.T., Ribeiro P.R.V., Silva L.M.A., Brito E.S., Canuto K.M. (2016). Resorcylic Lactones from *Lasiodiplodia Theobromae* (MUB65), a Fungal Endophyte Isolated from Myracrodruon Urundeuva. Planta Med..

[89] Yang X., Zhang L., Guo B., Guo S. (2004). Preliminary Study of a Vincristine-Proudcing Endophytic Fungus Isolated from Leaves of Catharanthus Roseus. Chin. Tradit. Herb. Drugs.

[90] Van der Sar S.A., Blunt J.W., Munro M.H.G. (2006). Spiro-Mamakone A:  A Unique Relative of the Spirobisnaphthalene Class of Compounds. Org. Lett..

[91] Moreno E., Varughese T., Spadafora C., Arnold A.E., Coley P.D., Kursar T.A., Gerwick W.H., Cubilla-Rios L. (2011). Chemical Constituents of the New Endophytic Fungus *Mycosphaerella* sp. Nov. and Their Anti-Parasitic Activity. Nat. Prod. Commun..

[92] Luo J., Liu X., Li E., Guo L., Che Y. (2013). Arundinols A–C and Arundinones A and B from the Plant Endophytic Fungus *Microsphaeropsis Arundinis*. J. Nat. Prod..

[93] Ortega H.E., Graupner P.R., Asai Y., TenDyke K., Qiu D., Shen Y.Y., Rios N., Arnold A.E., Coley P.D., Kursar T.A. (2013). Mycoleptodiscins A and B, Cytotoxic Alkaloids from the Endophytic Fungus *Mycoleptodiscus* sp. F0194. J. Nat. Prod..

[94] Lin T., Wang G., Shan W., Zeng D., Ding R., Jiang X., Zhu D., Liu X., Yang S., Chen H. (2014). Myrotheciumones: Bicyclic Cytotoxic Lactones Isolated from an Endophytic Fungus of Ajuga Decumbens. Bioorg. Med. Chem. Lett..

[95] Shen L., Zhu L., Tan Q., Wan D., Xie J., Peng J. (2016). New Cytotoxic Trichothecene Macrolide Epimers from Endophytic *Myrothecium Roridum* IFB-E012. J. Antibiot..

[96] Rehman S., Shawl A.S., Kour A., Andrabi R., Sudan P., Sultan P., Verma V., Qazi G.N. (2008). An Endophytic *Neurospora* sp. from Nothapodytes Foetida Producing Camptothecin. Appl. Biochem. Microbiol..

[97] Wu Z.-C., Li D.-L., Chen Y.-C., Zhang W.-M. (2010). A New Isofuranonaphthalenone and Benzopyrans from the Endophytic Fungus *Nodulisporium* sp. A4 from Aquilaria Sinensis. Helv. Chim. Acta.

[98] Borges Coutinho Gallo M., Coêlho Cavalcanti B., Washington Araújo Barros F., Odorico de Moraes M., Veras Costa-Lotufo L., Pessoa C., Kenupp Bastos J., Tallarico Pupo M. (2010). Chemical Constituents of *Papulaspora Immersa*, an Endophyte from Smallanthus Sonchifolius (Asteraceae), and Their Cytotoxic Activity. Chem. Biodivers..

[99] Shiono Y., Kikuchi M., Koseki T., Murayama T., Kwon E., Aburai N., Kimura K. (2011). Isopimarane Diterpene Glycosides, Isolated from Endophytic Fungus *Paraconiothyrium* sp. MY-42. Phytochemistry.

[100] Liu L., Chen X., Li D., Zhang Y., Li L., Guo L., Cao Y., Che Y. (2015). Bisabolane Sesquiterpenoids from the Plant Endophytic Fungus *Paraconiothyrium Brasiliense*. J. Nat. Prod..

[101] Huang Z., Yang J., Cai X., She Z., Lin Y. (2012). A New Furanocoumarin from the Mangrove Endophytic Fungus *Penicillium* sp. (ZH16). Nat. Prod. Res..

[102] Lin Z.-J., Lu Z.-Y., Zhu T.-J., Fang Y.-C., Gu Q.-Q., Zhu W.-M. (2008). Penicillenols from *Penicillium* sp. GQ-7, an Endophytic Fungus Associated with Aegiceras Corniculatum. Chem. Pharm. Bull..

[103] Lin Z., Zhu T., Fang Y., Gu Q., Zhu W. (2008). Polyketides from *Penicillium* sp. JP-1, an Endophytic Fungus Associated with the Mangrove Plant Aegiceras Corniculatum. Phytochemistry.

[104] Chen M.-J., Fu Y.-W., Zhou Q.-Y. (2014). Penifupyrone, a New Cytotoxic Funicone Derivative from the Endophytic Fungus *Penicillium* sp. HSZ-43. Nat. Prod. Res..

[105] Sun X., Kong X., Gao H., Zhu T., Wu G., Gu Q., Li D. (2014). Two New Meroterpenoids Produced by the Endophytic Fungus *Penicillium* sp. SXH-65. Arch. Pharm. Res..

[106] Darsih C., Prachyawarakorn V., Wiyakrutta S., Mahidol C., Ruchirawat S., Kittakoop P. (2015). Cytotoxic Metabolites from the Endophytic Fungus *Penicillium Chermesinum*: Discovery of a Cysteine-Targeted Michael Acceptor as a Pharmacophore for Fragment-Based Drug Discovery, Bioconjugation and Click Reactions. RSC Adv..

[107] El-Neketi M., Ebrahim W., Lin W., Gedara S., Badria F., Saad H.-E.A., Lai D., Proksch P. (2013). Alkaloids and Polyketides from *Penicillium Citrinum*, an Endophyte Isolated from the Moroccan Plant Ceratonia Siliqua. J. Nat. Prod..

[108] Ge H.-L., Zhang D.-W., Li L., Xie D., Zou J.-H., Si Y.-K., Dai J. (2011). Two New Terpenoids from Endophytic Fungus *Periconia* sp. F-31. Chem. Pharm. Bull..

[109] Teles H.L., Sordi R., Silva G.H., Castro-Gamboa I., da Silva Bolzani V., Pfenning L.H., de Abreu L.M., Costa-Neto C.M., Young M.C.M., Araújo Â.R. (2006). Aromatic Compounds Produced by *Periconia Atropurpurea*, an Endophytic Fungus Associated with Xylopia Aromatica. Phytochemistry.

[110] Xu J., Kjer J., Sendker J., Wray V., Guan H., Edrada R., Lin W., Wu J., Proksch P. (2009). Chromones from the Endophytic Fungus *Pestalotiopsis* sp. Isolated from the Chinese Mangrove Plant Rhizophora Mucronata. J. Nat. Prod..

[111] Davis R.A., Carroll A.R., Andrews K.T., Boyle G.M., Tran T.L., Healy P.C., Kalaitzis J.A., Shivas R.G. (2010). Pestalactams A–C: Novel Caprolactams from the Endophytic Fungus *Pestalotiopsis* sp.. Org. Biomol. Chem..

[112] Wu L.-S., Jia M., Chen L., Zhu B., Dong H.-X., Si J.-P., Peng W., Han T. (2015). Cytotoxic and Antifungal Constituents Isolated from the Metabolites of Endophytic Fungus DO14 from Dendrobium Officinale. Molecules.

[113] Liu S., Guo L., Che Y., Liu L. (2013). Pestaloficiols Q–S from the Plant Endophytic Fungus *Pestalotiopsis Fici*. Fitoterapia.

[114] LIU S.-C., YE X., GUO L.-D., LIU L. (2011). Cytotoxic Isoprenylated Epoxycyclohexanediols from the Plant Endophyte *Pestalotiopsis Fici*. Chin. J. Nat. Med..

[115] Liu L., Liu S., Niu S., Guo L., Chen X., Che Y. (2009). Isoprenylated Chromone Derivatives from the Plant Endophytic Fungus *Pestalotiopsis Fici*. J. Nat. Prod..

[116] Luo D.Q., Zhang L., Shi B.Z., Song X.M. (2012). Two New Oxysporone Derivatives from the Fermentation Broth of the Endophytic Plant Fungus *Pestalotiopsis Karstenii* Isolated from Stems of Camellia Sasanqua. Molecules.

[117] Kumaran R.S., Choi Y.-K., Lee S., Jeon H.J., Jung H., Kim H.J. (2012). Isolation of Taxol, an Anticancer Drug Produced by the Endophytic Fungus, *Phoma Betae*. Afr. J. Biotechnol..

[118] Rajendran L., Rajagopal K., Subbarayan K., Ulagappan K., Sampath A., Karthik G. (2013). Efficiency of Fungal Taxol on Human Liver Carcinoma Cell Lines. Am. J. Res. Commun..

[119] Lee J.C., Strobel G.A., Lobkovsky E., Clardy J. (1996). Torreyanic Acid:  A Selectively Cytotoxic Quinone Dimer from the Endophytic Fungus *Pestalotiopsis Microspora*. J. Org. Chem..

[120] Metz A.M., Haddad A., Worapong J., Long D.M., Ford E.J., Hess W.M., Strobel G.A. (2000). Induction of the Sexual Stage of *Pestalotiopsis Microspora*, a Taxol-Producing Fungus. Microbiology.

[121] Vennila R., Kamalraj S., Muthumary J. (2012). In Vitro Studies on Anticancer Activity of Fungal Taxol against Human Breast Cancer Cell Line MCF-7 Cells. Asian Pac. J. Trop. Biomed..

[122] Ding G., Qi Y., Liu S., Guo L., Chen X. (2012). Photipyrones A and B, New Pyrone Derivatives from the Plant Endophytic Fungus *Pestalotiopsis Photiniae*. J. Antibiot..

[123] Ding G., Zheng Z., Liu S., Zhang H., Guo L., Che Y. (2009). Photinides A–F, Cytotoxic Benzofuranone-Derived γ-Lactones from the Plant Endophytic Fungus *Pestalotiopsis Photiniae*. J. Nat. Prod..

[124] Nalli Y., Mirza D.N., Wani Z.A., Wadhwa B., Mallik F.A., Raina C., Chaubey A., Riyaz-Ul-Hassan S., Ali A. (2015). Phialomustin A–D, New Antimicrobial and Cytotoxic Metabolites from an Endophytic Fungus, *Phialophora Mustea*. RSC Adv..

[125] Santiago C., Sun L., Munro M.H.G., Santhanam J. (2014). Polyketide and Benzopyran Compounds of an Endophytic Fungus Isolated from C Innamomum Mollissimum: Biological Activity and Structure. Asian Pac. J. Trop. Biomed..

[126] Huang Z., Guo Z., Yang R., Yin X., Li X., Luo W., She Z., Lin Y. (2009). Chemistry and Cytotoxic Activities of Polyketides Produced by the Mangrove Endophytic Fungus *Phomopsis* SP. ZSU-H76. Chem. Nat. Compd..

[127] Huang Z., Yang J., Lei F., She Z., Lin Y. (2013). A New Xanthone O-Glycoside from the Mangrove Endophytic Fungus *Phomopsis* sp.. Chem. Nat. Compd..

[128] Zhang W., Xu L., Yang L., Huang Y., Li S., Shen Y. (2014). Phomopsidone A, a Novel Depsidone Metabolite from the Mangrove Endophytic Fungus *Phomopsis* sp. A123. Fitoterapia.

[129] Isaka M., Jaturapat A., Rukseree K., Danwisetkanjana K., Tanticharoen M., Thebtaranonth Y. (2001). Phomoxanthones A and B, Novel Xanthone Dimers from the Endophytic Fungus Phomopsis Species. J. Nat. Prod..

[130] Bunyapaiboonsri T., Yoiprommarat S., Srikitikulchai P., Srichomthong K., Lumyong S. (2010). Oblongolides from the Endophytic Fungus *Phomopsis* sp. BCC 9789. J. Nat. Prod..

[131] Jouda J.-B., Tamokou J.-D., Mbazoa C.D., Douala-Meli C., Sarkar P., Bag P.K., Wandji J. (2016). Antibacterial and Cytotoxic Cytochalasins from the Endophytic Fungus *Phomopsis* sp. Harbored in Garcinia Kola (Heckel) Nut. BMC Complement. Altern. Med..

[132] Wagenaar M.M., Clardy J. (2001). Dicerandrols, New Antibiotic and Cytotoxic Dimers Produced by the Fungus *Phomopsis Longicolla* Isolated from an Endangered Mint. J. Nat. Prod..

[133] Wijeratne E.K., Paranagama P.A., Marron M.T., Gunatilaka M.K., Arnold A.E., Gunatilaka A.L. (2008). Sesquiterpene Quinones and Related Metabolites from *Phyllosticta Spinarum*, a Fungal Strain Endophytic in Platycladus Orientalis of the Sonoran Desert (1). J. Nat. Prod..

[134] Deshmukh S.K., Mishra P.D., Kulkarni-Almeida A., Verekar S., Sahoo M.R., Periyasamy G., Goswami H., Khanna A., Balakrishnan A., Vishwakarma R. (2009). Anti-Inflammatory and Anticancer Activity of Ergoflavin Isolated from an Endophytic Fungus. Chem. Biodivers..

[135] Chen X., Shi Q., Lin G., Guo S., Yang J. (2009). Spirobisnaphthalene Analogues from the Endophytic Fungus *Preussia* sp.. J. Nat. Prod..

[136] Wagenaar M.M., Corwin J., Strobel G., Clardy J. (2000). Three New Cytochalasins Produced by an Endophytic Fungus in the Genus Rhinocladiella. J. Nat. Prod..

[137] Pudhom K., Teerawatananond T., Chookpaiboon S. (2014). Spirobisnaphthalenes from the Mangrove-Derived Fungus *Rhytidhysteron* sp. AS21B. Mar. Drugs.

[138] Lai D., Wang A., Cao Y., Zhou K., Mao Z., Dong X., Tian J., Xu D., Dai J., Peng Y. (2016). Bioactive Dibenzo-α-Pyrone Derivatives from the Endophytic Fungus *Rhizopycnis Vagum* Nitaf22. J. Nat. Prod..

[139] Siridechakorn I., Yue Z., Mittraphab Y., Lei X., Pudhom K. (2017). Identification of Spirobisnaphthalene Derivatives with Anti-Tumor Activities from the Endophytic Fungus *Rhytidhysteron Rufulum* AS21B. Bioorg. Med. Chem..

[140] El-Elimat T., Figueroa M., Raja H.A., Graf T.N., Swanson S.M., Falkinham J.O., Wani M.C., Pearce C.J., Oberlies N.H. (2015). Biosynthetically Distinct Cytotoxic Polyketides from *Setophoma Terrestris*. Eur. J. Org. Chem..

[141] Wang X.-N., Bashyal B.P., Wijeratne E.M.K., U’Ren J.M., Liu M.X., Gunatilaka M.K., Arnold A.E., Gunatilaka A.A.L. (2011). Smardaesidins A–G, Isopimarane and 20-Nor-Isopimarane Diterpenoids from *Smardaea* sp., a Fungal Endophyte of the Moss Ceratodon Purpureus. J. Nat. Prod..

[142] Mirjalili M.H., Farzaneh M., Bonfill M., Rezadoost H., Ghassempour A. (2012). Isolation and Characterization of *Stemphylium Sedicola* SBU-16 as a New Endophytic Taxol-Producing Fungus from Taxus Baccata Grown in Iran. FEMS Microbiol. Lett..

[143] Debbab A., Aly A.H., Edrada-Ebel R., Wray V., Müller W.E.G., Totzke F., Zirrgiebel U., Schächtele C., Kubbutat M.H.G., Lin W.H. (2009). Bioactive Metabolites from the Endophytic Fungus *Stemphylium Globuliferum* Isolated from Mentha Pulegium. J. Nat. Prod..

[144] Teiten M.-H., Mack F., Debbab A., Aly A.H., Dicato M., Proksch P., Diederich M. (2013). Anticancer Effect of Altersolanol A, a Metabolite Produced by the Endophytic Fungus *Stemphylium Globuliferum*, Mediated by Its pro-Apoptotic and Anti-Invasive Potential via the Inhibition of NF-ΚB Activity. Bioorg. Med. Chem..

[145] Zhao Q.-H., Yang Z.-D., Shu Z.-M., Wang Y.-G., Wang M.-G. (2016). Secondary Metabolites and Biological Activities of *Talaromyces* sp. LGT-2, an Endophytic Fungus from Tripterygium Wilfordii. Iran. J. Pharm. Res. IJPR.

[146] Li H., Huang H., Shao C., Huang H., Jiang J., Zhu X., Liu Y., Liu L., Lu Y., Li M. (2011). Cytotoxic Norsesquiterpene Peroxides from the Endophytic Fungus *Talaromyces Flavus* Isolated from the Mangrove Plant Sonneratia Apetala. J. Nat. Prod..

[147] Kusari S., Zühlke S., Košuth J., Čellárová E., Spiteller M. (2009). Light-Independent Metabolomics of Endophytic *Thielavia Subthermophila* Provides Insight into Microbial Hypericin Biosynthesis. J. Nat. Prod..

[148] Puri S.C., Nazir A., Chawla R., Arora R., Riyaz-ul-Hasan S., Amna T., Ahmed B., Verma V., Singh S., Sagar R. (2006). The Endophytic Fungus *Trametes Hirsuta* as a Novel Alternative Source of Podophyllotoxin and Related Aryl Tetralin Lignans. J. Biotechnol..

[149] Ding G., Wang H., Li L., Chen A.J., Chen L., Chen H., Zhang H., Liu X., Zou Z. (2012). Trichoderones A and B: Two Pentacyclic Cytochalasans from the Plant Endophytic Fungus *Trichoderma Gamsii*. Eur. J. Org. Chem..

[150] Taware R., Abnave P., Patil D., Rajamohananan P.R., Raja R., Soundararajan G., Kundu G.C., Ahmad A. (2014). Isolation, Purification and Characterization of Trichothecinol-A Produced by Endophytic Fungus *Trichothecium* sp. and Its Antifungal, Anticancer and Antimetastatic Activities. Sustain. Chem. Process..

[151] Chokpaiboon S., Sommit D., Teerawatananond T., Muangsin N., Bunyapaiboonsri T., Pudhom K. (2010). Cytotoxic Nor-Chamigrane and Chamigrane Endoperoxides from a Basidiomycetous Fungus. J. Nat. Prod..

[152] Isaka M., Chinthanom P., Boonruangprapa T., Rungjindamai N., Pinruan U. (2010). Eremophilane-Type Sesquiterpenes from the Fungus *Xylaria* sp. BCC 21097. J. Nat. Prod..

[153] Tansuwan S., Pornpakakul S., Roengsumran S., Petsom A., Muangsin N., Sihanonta P., Chaichit N. (2007). Antimalarial Benzoquinones from an Endophytic Fungus, *Xylaria* sp.. J. Nat. Prod..

[154] Wei H., Xu Y., Espinosa-Artiles P., Liu M.X., Luo J.-G., U’Ren J.M., Elizabeth Arnold A., Leslie Gunatilaka A.A. (2015). Sesquiterpenes and Other Constituents of *Xylaria* sp. NC1214, a Fungal Endophyte of the Moss *Hypnum* sp.. Phytochemistry.

[155] Zhang Q., Xiao J., Sun Q.-Q., Qin J.-C., Pescitelli G., Gao J.-M. (2014). Characterization of Cytochalasins from the Endophytic *Xylaria* sp. and Their Biological Functions. J. Agric. Food Chem..

[156] Sawadsitang S., Mongkolthanaruk W., Suwannasai N., Sodngam S. (2015). Antimalarial and Cytotoxic Constituents of *Xylaria Cf. Cubensis* PK108. Nat. Prod. Res..

[157] Lin T., Lin X., Lu C.-H., Shen Y.-M. (2011). Three New Triterpenes from *Xylarialean* sp. A45, an Endophytic Fungus from *Annona Squamosa* L.. Helv. Chim. Acta.

[158] Zhang J., Tao L., Liang Y., Yan Y., Dai C., Xia X., She Z., Lin Y., Fu L. (2009). Secalonic Acid D Induced Leukemia Cell Apoptosis and Cell Cycle Arrest of G(1) with Involvement of GSK-3beta/Beta-Catenin/c-Myc Pathway. Cell Cycle Georget. Tex.

[159] Kamdem R.S.T., Wang H., Wafo P., Ebrahim W., Özkaya F.C., Makhloufi G., Janiak C., Sureechatchaiyan P., Kassack M.U., Lin W. (2018). Induction of New Metabolites from the Endophytic Fungus Bionectria sp. through Bacterial Co-Culture. Fitoterapia.

[160] Ibrahim S.R.M., Mohamed G.A., Al Haidari R.A., Zayed M.F., El-Kholy A.A., Elkhayat E.S., Ross S.A. (2018). Fusarithioamide B, a New Benzamide Derivative from the Endophytic Fungus Fusarium Chlamydosporium with Potent Cytotoxic and Antimicrobial Activities. Bioorg. Med. Chem..

[161] Zhang X., Liu J., Tang P., Liu Z., Guo G.-J., Sun Q.-Y., Yin J. (2018). Identification of a New Uncompetitive Inhibitor of Adenosine Deaminase from Endophyte *Aspergillus Niger* sp.. Curr. Microbiol..

[162] Tan X.-M., Li L.-Y., Sun L.-Y., Sun B.-D., Niu S.-B., Wang M.-H., Zhang X.-Y., Sun W.-S., Zhang G.-S., Deng H. (2018). Spiciferone Analogs from an Endophytic Fungus *Phoma Betae* Collected from Desert Plants in West China. J. Antibiot..

[163] Liu Z., Zhao J.-Y., Li Y., Lyu X.-X., Liu Y.-B. (2018). Investigations on secondary metabolites of endophyte *Diaporthe* sp. hosted in Tylophora ovata. Zhongguo Zhong Yao Za Zhi Zhongguo Zhongyao Zazhi China J. Chin. Mater. Medica.

[164] Sharma V., Singamaneni V., Sharma N., Kumar A., Arora D., Kushwaha M., Bhushan S., Jaglan S., Gupta P. (2018). Valproic Acid Induces Three Novel Cytotoxic Secondary Metabolites in *Diaporthe* sp., an Endophytic Fungus from Datura Inoxia Mill. Bioorg. Med. Chem. Lett..

[165] Sharma N., Kushwaha M., Arora D., Jain S., Singamaneni V., Sharma S., Shankar R., Bhushan S., Gupta P., Jaglan S. (2018). New Cytochalasin from Rosellinia Sanctae-Cruciana, an Endophytic Fungus of Albizia Lebbeck. J. Appl. Microbiol..

[166] Kamdem R.S.T., Pascal W., Rehberg N., van Geelen L., Höfert S.-P., Knedel T.-O., Janiak C., Sureechatchaiyan P., Kassack M.U., Lin W. (2018). Metabolites from the Endophytic Fungus *Cylindrocarpon* sp. Isolated from Tropical Plant Sapium Ellipticum. Fitoterapia.

[167] Vu H.-N.T., Nguyen D.T., Nguyen H.Q., Chu H.H., Chu S.K., Chau M.V., Phi Q.-T. (2018). Antimicrobial and Cytotoxic Properties of Bioactive Metabolites Produced by Streptomyces Cavourensis YBQ59 Isolated from Cinnamomum Cassia Prels in Yen Bai Province of Vietnam. Curr. Microbiol..

[168] Liu H.-X., Tan H.-B., Chen Y.-C., Li S.-N., Li H.-H., Zhang W.-M. (2018). Secondary Metabolites from the *Colletotrichum Gloeosporioides* A12, an Endophytic Fungus Derived from Aquilaria Sinensis. Nat. Prod. Res..

[169] Ouyang J., Mao Z., Guo H., Xie Y., Cui Z., Sun J., Wu H., Wen X., Wang J., Shan T. (2018). Mollicellins O–R, Four New Depsidones Isolated from the Endophytic Fungus *Chaetomium* sp. Eef-10. Molecules.

[170] Zhou J., Li G., Deng Q., Zheng D., Yang X., Xu J. (2018). Cytotoxic Constituents from the Mangrove Endophytic *Pestalotiopsis* sp. Induce G0/G1 Cell Cycle Arrest and Apoptosis in Human Cancer Cells. Nat. Prod. Res..

[171] Ariantari N.P., Ancheeva E., Wang C., Mándi A., Knedel T.-O., Kurtán T., Chaidir C., Müller W.E.G., Kassack M.U., Janiak C. (2019). Indole Diterpenoids from an Endophytic *Penicillium* sp.. J. Nat. Prod..

[172] Senthil Kumar V., Kumaresan S., Tamizh M.M., Hairul Islam M.I., Thirugnanasambantham K. (2019). Anticancer Potential of NF-ΚB Targeting Apoptotic Molecule “Flavipin” Isolated from Endophytic *Chaetomium Globosum*. Phytomedicine.

[173] Wang W.-X., Zheng M.-J., Li J., Feng T., Li Z.-H., Huang R., Zheng Y.-S., Sun H., Ai H.-L., Liu J.-K. (2019). Cytotoxic Polyketides from Endophytic Fungus Phoma Bellidis Harbored in Ttricyrtis Maculate. Phytochem. Lett..

[174] Harwoko H., Daletos G., Stuhldreier F., Lee J., Wesselborg S., Feldbrügge M., Müller W.E.G., Kalscheuer R., Ancheeva E., Proksch P. (2021). Dithiodiketopiperazine Derivatives from Endophytic Fungi Trichoderma Harzianum and Epicoccum Nigrum. Nat. Prod. Res..

[175] Xin X.-Q., Chen Y., Zhang H., Li Y., Yang M.-H., Kong L.-Y. (2019). Cytotoxic Seco-Cytochalasins from an Endophytic *Aspergillus* sp. Harbored in Pinellia Ternata Tubers. Fitoterapia.

[176] Wang F., Zhao W., Zhang C., Chang S., Shao R., Xing J., Chen M., Zhang Y., Si S. (2019). Cytotoxic Metabolites from the Endophytic Fungus *Chaetomium Globosum* 7951. RSC Adv..

[177] Peng F., Hou S.-Y., Zhang T.-Y., Wu Y.-Y., Zhang M.-Y., Yan X.-M., Xia M.-Y., Zhang Y.-X. (2019). Cytotoxic and Antimicrobial Indole Alkaloids from an Endophytic Fungus *Chaetomium* sp. SYP-F7950 of Panax Notoginseng. RSC Adv..

[178] Chen Y., Liu Z., Huang Y., Liu L., He J., Wang L., Yuan J., She Z. (2019). Ascomylactams A–C, Cytotoxic 12- or 13-Membered-Ring Macrocyclic Alkaloids Isolated from the Mangrove Endophytic Fungus *Didymella* sp. CYSK-4, and Structure Revisions of Phomapyrrolidones A and C. J. Nat. Prod..

[179] Li G., Xu K., Chen W.-Q., Guo Z.-H., Liu Y.-T., Qiao Y.-N., Sun Y., Sun G., Peng X.-P., Lou H.-X. (2019). Heptaketides from the Endophytic Fungus *Pleosporales* sp. F46 and Their Antifungal and Cytotoxic Activities. RSC Adv..

[180] Kumarihamy M., Ferreira D., Croom E.M., Sahu R., Tekwani B.L., Duke S.O., Khan S., Techen N., Nanayakkara N.P.D. (2019). Antiplasmodial and Cytotoxic Cytochalasins from an Endophytic Fungus, *Nemania* sp. UM10M, Isolated from a Diseased Torreya Taxifolia Leaf. Molecules.

[181] He W., Xu Y., Fu P., Zuo M., Liu W., Jiang Y., Wang L., Zhu W. (2019). Cytotoxic Indolyl Diketopiperazines from the *Aspergillus* sp. GZWMJZ-258, Endophytic with the Medicinal and Edible Plant Garcinia Multiflora. J. Agric. Food Chem..

[182] Wang W.-X., Li Z.-H., Ai H.-L., Li J., He J., Zheng Y.-S., Feng T., Liu J.-K. (2019). Cytotoxic 19,20-Epoxycytochalasans from Endophytic Fungus Xylaria Cf. Curta. Fitoterapia.

[183] De Amorim M.R., Hilário F., Junior F.M. (2019). dos S.; Junior, J.M.B.; Bauab, T.M.; Araújo, A.R.; Carlos, I.Z.; Vilegas, W.; Santos, L.C. dos New Benzaldehyde and Benzopyran Compounds from the Endophytic Fungus *Paraphaeosphaeria* sp. F03 and Their Antimicrobial and Cytotoxic Activities. Planta Med..

[184] Zhao T., Xu L.-L., Zhang Y., Lin Z.-H., Xia T., Yang D.-F., Chen Y.-M., Yang X.-L. (2019). Three New α-Pyrone Derivatives from the Plant Endophytic Fungus Penicillium Ochrochloronthe and Their Antibacterial, Antifungal, and Cytotoxic Activities. J. Asian Nat. Prod. Res..

[185] Xu J., Hu Y.-W., Qu W., Chen M.-H., Zhou L.-S., Bi Q.-R., Luo J.-G., Liu W.-Y., Feng F., Zhang J. (2019). Cytotoxic and Neuroprotective Activities of Constituents from Alternaria Alternate, a Fungal Endophyte of Psidium Littorale. Bioorg. Chem..

[186] Wu Y., Chen S., Liu H., Huang X., Liu Y., Tao Y., She Z. (2019). Cytotoxic Isocoumarin Derivatives from the Mangrove Endophytic Fungus *Aspergillus* sp. HN15-5D. Arch. Pharm. Res..

[187] Fu J., Hu L., Shi Z., Sun W., Yue D., Wang Y., Ma X., Ren Z., Zuo Z., Peng G. (2021). Two Metabolites Isolated from Endophytic Fungus *Coniochaeta* sp. F-8 in Ageratina Adenophora Exhibit Antioxidative Activity and Cytotoxicity. Nat. Prod. Res..

[188] Nalli Y., Arora P., Khan S., Malik F., Riyaz-Ul-Hassan S., Gupta V., Ali A. (2019). Isolation, Structural Modification of Macrophin from Endophytic Fungus Phoma Macrostoma and Their Cytotoxic Potential. Med. Chem. Res..

[189] Shen L., Ai C.-Z., Song Y.-C., Wang F.-W., Jiao R.-H., Zhang A.-H., Man H.-Z., Tan R.-X. (2019). Cytotoxic Trichothecene Macrolides Produced by the Endophytic *Myrothecium Roridum*. J. Nat. Prod..

[190] Gubiani J.R., Oliveira M.C.S., Neponuceno R.A.R., Camargo M.J., Garcez W.S., Biz A.R., Soares M.A., Araujo A.R., da Bolzani V.S., Lisboa H.C.F. (2019). Cytotoxic Prenylated Indole Alkaloid Produced by the Endophytic Fungus *Aspergillus Terreus* P63. Phytochem. Lett..

[191] Rao L., You Y.-X., Su Y., Liu Y., He Q., Fan Y., Hu F., Xu Y.-K., Zhang C.-R. (2019). Two Spiroketal Derivatives with an Unprecedented Amino Group and Their Cytotoxicity Evaluation from the Endophytic Fungus Pestalotiopsis Flavidula. Fitoterapia.

[192] Zhang H., Yang M.-H., Zhuo F., Gao N., Cheng X.-B., Wang X.-B., Pei Y.-H., Kong L.-Y. (2019). Seven New Cytotoxic Phenylspirodrimane Derivatives from the Endophytic Fungus Stachybotrys Chartarum. RSC Adv..

[193] Li X.-H., Han X.-H., Qin L.-L., He J.-L., Cao Z.-X., Gu Y.-C., Guo D.-L., Deng Y. (2019). Isochromanes from *Aspergillus Fumigatus*, an Endophytic Fungus from Cordyceps Sinensis. Nat. Prod. Res..

[194] Elissawy A.M., Ebada S.S., Ashour M.L., El-Neketi M., Ebrahim W., Singab A.B. (2019). New Secondary Metabolites from the Mangrove-Derived Fungus *Aspergillus* sp. AV-2. Phytochem. Lett..

[195] Zhang H.-M., Ju C.-X., Li G., Sun Y., Peng Y., Li Y.-X., Peng X.-P., Lou H.-X. (2019). Dimeric 1,4-Benzoquinone Derivatives with Cytotoxic Activities from the Marine-Derived Fungus *Penicillium* sp. L129. Mar. Drugs.

[196] Yang B., Tong Q., Lin S., Guo J., Zhang J., Liu J., Wang J., Zhu H., Hu Z., Zhang Y. (2019). Cytotoxic Butenolides and Diphenyl Ethers from the Endophytic Fungus *Pestalotiopsis* sp.. Phytochem. Lett..

[197] Li Q., Chen C., Cheng L., Wei M., Dai C., He Y., Gong J., Zhu R., Li X.-N., Liu J. (2019). Emeridones A–F, a Series of 3,5-Demethylorsellinic Acid-Based Meroterpenoids with Rearranged Skeletons from an Endophytic Fungus *Emericella* sp. TJ29. J. Org. Chem..

[198] Liu H., Chen Y., Li H., Li S., Tan H., Liu Z., Li D., Liu H., Zhang W. (2019). Four New Metabolites from the Endophytic Fungus Diaporthe Lithocarpus A740. Fitoterapia.

[199] Narmani A., Teponno R.B., Arzanlou M., Surup F., Helaly S.E., Wittstein K., Praditya D.F., Babai-Ahari A., Steinmann E., Stadler M. (2019). Cytotoxic, Antimicrobial and Antiviral Secondary Metabolites Produced by the Plant Pathogenic Fungus *Cytospora* sp. CCTU A309. Fitoterapia.

[200] Cheng M.-J., Yang S.-S., Wu M.-D., Chang H.-H., Kuo Y.-H., Hsieh S.-Y., Chen J.-J., Wu H.-C. (2019). Isolation and Structure Elucidation of Secondary Metabolites From an Endophytic Fungus Annulohypoxylon Ilanense. Nat. Prod. Commun..

[201] Liu S., Zhao Y., Heering C., Janiak C., Müller W.E.G., Akoné S.H., Liu Z., Proksch P. (2019). Sesquiterpenoids from the Endophytic Fungus Rhinocladiella Similis. J. Nat. Prod..

[202] Chen S., Li H., Chen Y., Li S., Xu J., Guo H., Liu Z., Zhu S., Liu H., Zhang W. (2019). Three New Diterpenes and Two New Sesquiterpenoids from the Endophytic Fungus Trichoderma Koningiopsis A729. Bioorg. Chem..

[203] Wang W.-X., Feng T., Li Z.-H., Li J., Ai H.-L., Liu J.-K. (2019). Cytochalasins D1 and C1, Unique Cytochalasans from Endophytic Fungus Xylaria Cf. Curta. Tetrahedron Lett..

[204] Long Y., Tang T., Wang L.-Y., He B., Gao K. (2019). Absolute Configuration and Biological Activities of Meroterpenoids from an Endophytic Fungus of Lycium Barbarum. J. Nat. Prod..

[205] Barakat F., Vansteelandt M., Triastuti A., Jargeat P., Jacquemin D., Graton J., Mejia K., Cabanillas B., Vendier L., Stigliani J.-L. (2019). Thiodiketopiperazines with Two Spirocyclic Centers Extracted from Botryosphaeria Mamane, an Endophytic Fungus Isolated from *Bixa Orellana* L.. Phytochemistry.

[206] Guo L., Lin J., Niu S., Liu S., Liu L. (2020). Pestalotiones A–D: Four New Secondary Metabolites from the Plant Endophytic Fungus Pestalotiopsis Theae. Molecules.

[207] Yu X., Müller W.E.G., Meier D., Kalscheuer R., Guo Z., Zou K., Umeokoli B.O., Liu Z., Proksch P. (2020). Polyketide Derivatives from Mangrove Derived Endophytic Fungus Pseudopestalotiopsis Theae. Mar. Drugs.

[208] Abdou R., Shabana S., Rateb M.E. (2020). Terezine E, Bioactive Prenylated Tryptophan Analogue from an Endophyte of Centaurea Stoebe. Nat. Prod. Res..

[209] de Oliveira Filho J.W.G., Andrade T.d.J.A.d.S., de Lima R.M.T., Silva D.H.S., dos Reis A.C., Santos J.V.d.O., de Meneses A.-A.P.M., de Carvalho R.M., da Mata A.M.O., de Alencar M.V.O.B. (2020). Cytogenotoxic Evaluation of the Acetonitrile Extract, Citrinin and Dicitrinin-A from *Penicillium Citrinum*. Drug Chem. Toxicol..

[210] Elsbaey M., Tanaka C., Miyamoto T. (2020). Allantopyrone E, a Rare α-Pyrone Metabolite from the Mangrove Derived Fungus Aspergillus Versicolor. Nat. Prod. Res..

[211] Wei C., Deng Q., Sun M., Xu J. (2020). Cytospyrone and Cytospomarin: Two New Polyketides Isolated from Mangrove Endophytic Fungus, *Cytospora* sp.. Molecules.

[212] Deng M., Tao L., Qiao Y., Sun W., Xie S., Shi Z., Qi C., Zhang Y. (2020). New Cytotoxic Secondary Metabolites against Human Pancreatic Cancer Cells from the Hypericum Perforatum Endophytic Fungus *Aspergillus Terreus*. Fitoterapia.

[213] Li X.-Q., Dong Q.-J., Xu K., Yuan X.-L., Liu X.-M., Zhang P. (2020). Cytotoxic Xanthones from the Plant Endophytic Fungus *Paecilamyces* sp. TE-540. Nat. Prod. Res..

[214] Wen S., Fan W., Guo H., Huang C., Yan Z., Long Y. (2020). Two New Secondary Metabolites from the Mangrove Endophytic Fungus *Pleosporales* sp. SK7. Nat. Prod. Res..

[215] Wang A., Yin R., Zhou Z., Gu G., Dai J., Lai D., Zhou L. (2020). Eremophilane-Type Sesquiterpenoids From the Endophytic Fungus *Rhizopycnis Vagum* and Their Antibacterial, Cytotoxic, and Phytotoxic Activities. Front. Chem..

[216] Gao Y., Stuhldreier F., Schmitt L., Wesselborg S., Guo Z., Zou K., Mándi A., Kurtán T., Liu Z., Proksch P. (2020). Induction of New Lactam Derivatives From the Endophytic Fungus Aplosporella Javeedii Through an OSMAC Approach. Front. Microbiol..

[217] Liu H., Chen Y., Li S., Zhang W., Liu Z., Tan H., Zhang W. (2020). Trichothecene Macrolides from the Endophytic Fungus Para*myrothecium Roridum* and Their Cytotoxic Activity. Fitoterapia.

[218] Bang S., Kwon H.E., Baek J.Y., Jang D.S., Kim S., Nam S.-J., Lee D., Kang K.S., Shim S.H. (2020). Colletotrichalactones A-Ca, Unusual 5/6/10-Fused Tricyclic Polyketides Produced by an Endophytic Fungus, *Colletotrichum* sp. JS-0361. Bioorganic Chem..

[219] Riga R., Happyana N., Quentmeier A., Zammarelli C., Kayser O., Hakim E.H. (2021). Secondary Metabolites from Diaporthe Lithocarpus Isolated from Artocarpus Heterophyllus. Nat. Prod. Res..

[220] Yu S., Zhu Y.-X., Peng C., Li J. (2021). Two New Sterol Derivatives Isolated from the Endophytic Fungus Aspergillus Tubingensis YP-2. Nat. Prod. Res..

[221] Chen L., Zhang Q.-Y., Jia M., Ming Q.-L., Yue W., Rahman K., Qin L.-P., Han T. (2014). Endophytic Fungi with Antitumor Activities: Their Occurrence and Anticancer Compounds. Crit. Rev. Microbiol..

[222] Newman D.J., Cragg G.M. (2016). Natural Products as Sources of New Drugs from 1981 to 2014. J. Nat. Prod..

[223] Cragg G.M., Newman D.J. (2005). Plants as a Source of Anti-Cancer Agents. J. Ethnopharmacol..

[224] Ling-hua M., Zhi-yong L., Pommier Y. (2003). Non-Camptothecin DNA Topoisomerase I Inhibitors in Cancer Therapy. Curr. Top. Med. Chem..

[225] Pommier Y. (2006). Topoisomerase I Inhibitors: Camptothecins and Beyond. Nat. Rev. Cancer.

[226] Haidle A.M., Myers A.G. (2004). An Enantioselective, Modular, and General Route to the Cytochalasins: Synthesis of L-696,474 and Cytochalasin B. Proc. Natl. Acad. Sci. USA.

[227] Svoboda G. (1961). Alkaloids of Vinca Rosea (Catharanthus Roseus). IX. Extraction and Characterization of Leurosidine and Leurocristine. Subj. Strain Bibliogr..

[228] Kawada M., Inoue H., Ohba S.-I., Masuda T., Momose I., Ikeda D. (2010). Leucinostatin A Inhibits Prostate Cancer Growth through Reduction of Insulin-like Growth Factor-I Expression in Prostate Stromal Cells. Int. J. Cancer.

[229] Chowdhury N.S., Sohrab H., Rana S., Hasan C.M., Jamshidi S., Rahman K.M. (2017). Cytotoxic Naphthoquinone and Azaanthraquinone Derivatives from an Endophytic *Fusarium Solani*. J. Nat. Prod..

[230] Kharwar R.N., Mishra A., Gond S.K., Stierle A., Stierle D. (2011). Anticancer Compounds Derived from Fungal Endophytes; Their Importance and Future Challenges. Nat. Prod. Rep..

[231] Wani M.C., Taylor H.L., Wall M.E., Coggon P., McPhail A.T. (1971). Plant Antitumor Agents. VI. Isolation and Structure of Taxol, a Novel Antileukemic and Antitumor Agent from *Taxus Brevifolia*. J. Am. Chem. Soc..

[232] Cragg G.M., Kingston D.G.I., Newman D.J. (2012). Anticancer Agents from Natural Products.

[233] Zhang P., Li X., Yuan X.-L., Du Y.-M., Wang B.-G., Zhang Z.-F. (2018). Antifungal Prenylated Diphenyl Ethers from Arthrinium Arundinis, an Endophytic Fungus Isolated from the Leaves of Tobacco (*Nicotiana Tabacum* L.). Molecules.

[234] Gao Y., Stuhldreier F., Schmitt L., Wesselborg S., Wang L., Müller W.E.G., Kalscheuer R., Guo Z., Zou K., Liu Z. (2020). Sesterterpenes and Macrolide Derivatives from the Endophytic Fungus Aplosporella Javeedii. Fitoterapia.

